# Molecular Classification of Lobular Carcinoma of the Breast

**DOI:** 10.1038/srep43265

**Published:** 2017-03-17

**Authors:** Denggang Fu, Qi Zuo, Qi Huang, Li Su, Huijun Z. Ring, Brian Z. Ring

**Affiliations:** 1 Institute of Genomic and Personalized Medicine, College of Life Science, Huazhong University of Science and Technology, Wuhan, Hubei 430074, China; 2 Key Laboratory of Molecular Biophysics, Ministry of Education, College of Life Science, Huazhong University of Science and Technology, Wuhan, Hubei 430074, China

## Abstract

The morphology of breast tumors is complicated and diagnosis can be difficult. We present here a novel diagnostic model which we validate on both array-based and RNA sequencing platforms which reliably distinguishes this tumor type across multiple cohorts. We also examine how this molecular classification predicts sensitivity to common chemotherapeutics in cell-line based assays. A total of 1845 invasive breast cancer cases in six cohorts were collected, split into discovery and validation cohorts, and a classifier was created and compared to pathological diagnosis, grade and survival. In the validation cohorts the concordance of predicted diagnosis with a pathological diagnosis was 92%, and 97% when inconclusively classified cases were excluded. Tumor-derived cell lines were classified with the model as having predominantly ductal or lobular-like molecular physiologies, and sensitivity of these lines to relevant compounds was analyzed. A diagnostic tool can be created that reliably distinguishes lobular from ductal carcinoma and allows the classification of cell lines on the basis of molecular profiles associated with these tumor types. This tool may assist in improved diagnosis and aid in explorations of the response of lobular type breast tumor models to different compounds.

Breast cancer is one of the most prevalent and malignant tumors, and is the second leading cause of cancer mortality among women^1^,^2^. A growing body of work has found that addressing its molecular and clinical heterogeneity is necessary to determine effective treatment strategies^3^,^4^. Invasive ductal breast carcinoma (IDC, or invasive carcinoma of no special type in the current WHO classification) and invasive lobular breast carcinoma (ILC) are the main two pathologically defined groups of mammary malignances, accounting for 80% and 15% of all the invasive breast cancer, respectively^5^,^6^,^7^,^8^,^9^. Additionally, several other pathologic variants have been reported in recent years^10^. Owing to its distinct histological patterns, biological and prognostic features, ILCs are generally believed to represent a specific entity, distinct from IDCs in terms of lineage and molecular features^11^,^12^,^13^,^14^, though the nature of this lineage is still a subject of active research.

Invasive ductal tumors are currently defined as a diagnosis of exclusion, not belonging to other breast subtypes, and accounts for the majority of breast cancer cases. These tumors tend to form glandular structures, whereas lobular tumors are generally confined to the terminal lobules, though can invade along the ductal system, and are less cohesive than ductal tumors^15^. Additionally, lobular tumors tend to be less aggressive than ductal tumors, and are frequently distinguished by their molecular physiology, as they often exhibit loss of E-cadherin and are typically estrogen and progesterone receptor positive^16^,^17^,^18^,^19^. Additionally, lobular tumors have been noted to have several distinctive genomic alterations, including gain on 1q and loss of chromosome 16q^20^. A recent large study of lobular features also categorized several mutations in the PTEN, TBX3, and FOXA1 genes that typify lobular carcinomas^14^. Additionally, ductal tumors often metastasize to the bone, lung and liver, via lymph glands and blood system, while lobular tumors are inclined to metastasize to genital tracts, the gastrointestinal system, and meninges^10^,^21^,^22^,^23^,^24^,^25^,^26^.

These observational studies suggest that ductal and lobular tumors’ progression and development follow distinctive pathways. The traditional theory of ductal and lobular breast carcinoma tumorigenesis supposed ductal carcinomas progress from ductal hyperplasias to ductal carcinoma *in situs* and ultimately to invade surrounding tissue, migrating into the fatty tissue after penetrating the wall of the ducts, while lobular carcinomas originate from lobular hyperplasias and progress to infiltrating lobular carcinomas. However, genomic studies cast doubt on this explanation, and suggest more complex developmental pathways^27^. Lobular carcinoma share some similarities with low-grade invasive ductal carcinoma, though with unique metastatic and histologic features, and it has been observed that many subtypes of mammary carcinoma appear to present genomic alterations or expression patterns strongly associated with histologic grade^27^,^28^,^29^,^30^. Pathologically defined lobular and ductal tumors when both are present in individual cases have been proposed to share clonal origins^31^. If true, then a reliable diagnosis depends on more than pathological clues to accurately differentiate these types of breast carcinomas.

Accurate diagnosis of breast tumor types is of increasing clinical relevance, and breast carcinoma histopathology is standardly used in helping predict prognostic outcome and devising treatment strategies. Lobular cancer may be less responsive to neoadjuvant chemotherapy^32^,^33^,^34^,^35^, even when adjusted for stage^36^. However, these tumors may respond similarly when treated at the adjuvant level, suggesting that factors beyond tumor cell drug sensitivity may play a role in defining the different responses^37^. Gene expression profiling has become a common tool to distinguish subtypes of breast cancer with clinical relevance^38^,^39^,^40^,^41^,^42^,^43^. However there are few studies which have proposed and validated a molecular assay that could distinguish ILCs from IDCs, especially as an intended clinical application. In this study, we aim to determine a robust set of genes whose expression can be used to differentiate between invasive lobular and ductal carcinomas. We propose a molecular model consisting of 46 genes that can reliably distinguish these tumor types and whose predicted diagnoses are more consistent with outcome than pathological diagnosis. Finally, cell line chemical sensitivity data is used to explore the relationship between predicted histopathological type and drug response in tumor models.

## Results

**Figure 1:**
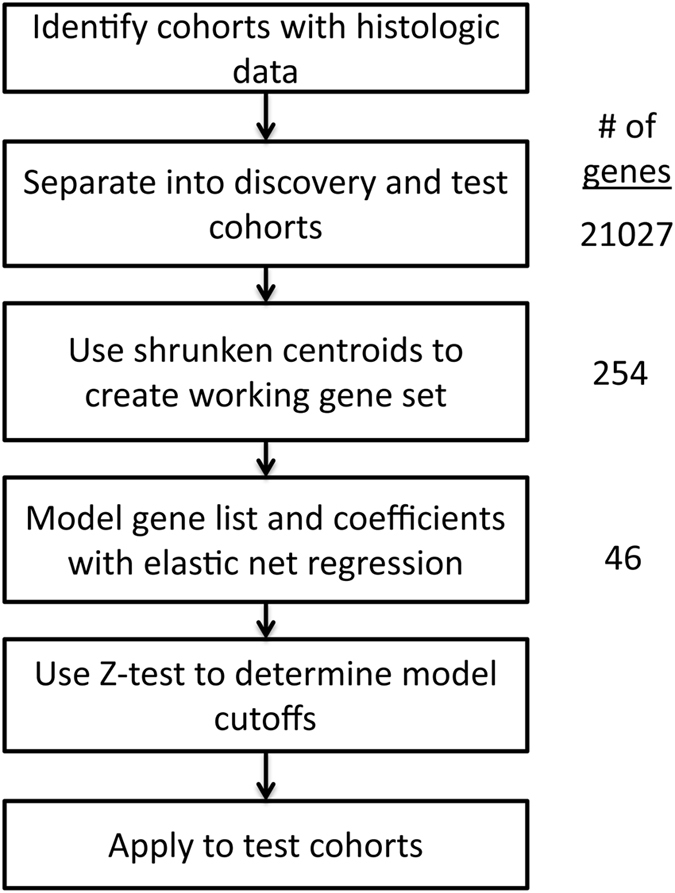
Flow chart of analysis.

A general analysis workflow is diagrammed in Fig. 1. Gene expression data from six independent cohorts were acquired for which pathologically determined lobular and ductal carcinomas had been determined, consisting of 1845 samples in total (Table 1), with three cohorts set as a discovery group (N = 653) and three cohorts for validations (N = 1192). Tumors of lobular origin accounted for 7.8–16.8% of the samples in these cohorts. Gene expression had been measured on these samples using printed oligo arrays (Affymetrix, Agilent) and RNA sequencing.

**Figure 2:**
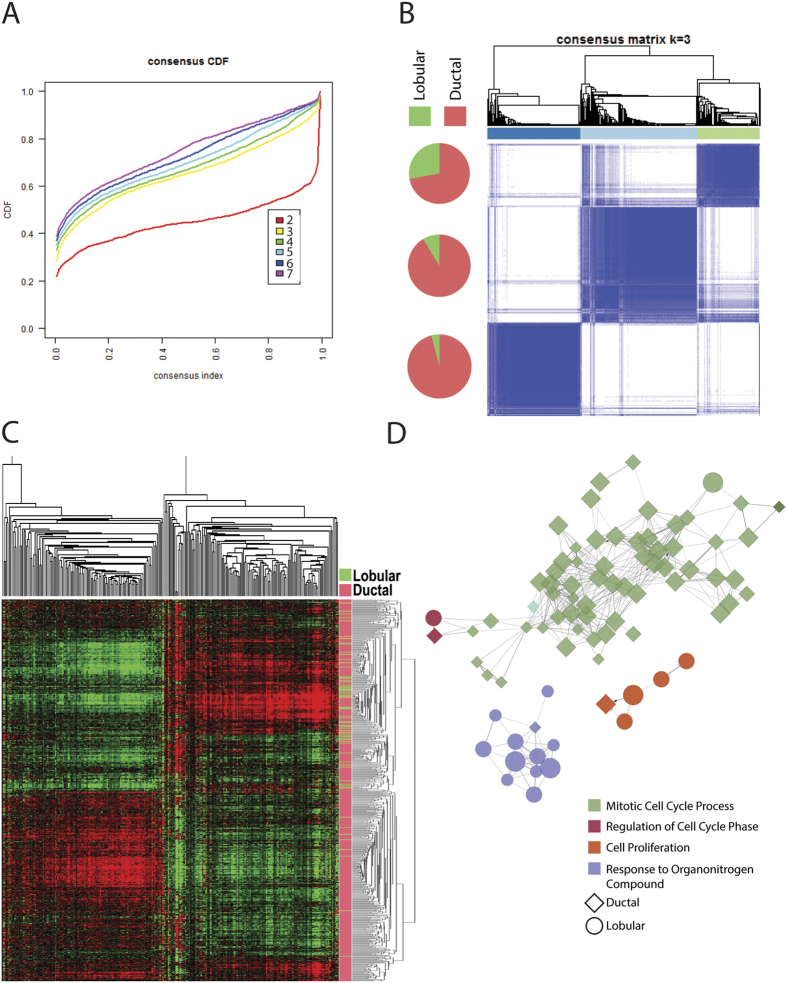
Lobular and ductal histologic classes and gene expression. (**A**) The cumulative distribution from consensus matrices for clustering with 2 to 7 clusters. (**B**) Consensus clustering with k = 3. Fraction of ductal and lobular cases is displayed. (**C**) Clustering of 250 shrunken centroid selected genes. Vertical axis: tumor samples, horizontal axis: genes. (**D**) Network of gene ontology terms associated with shrunken centroid –defined classifying genes. Go terms are functionally grouped and color coded. GO terms whose associated genes are more prevalent in the lobular classifiers are circular, those with more ductal classifying associated genes are diamond shaped.

To understand how overall lobular and ductal phenotypes relate to the underlying molecular physiology of the tumors, and to assess diversity within these histological types, we calculated the cumulative distribution from consensus matrices for clustering with 2 to 7 clusters. Consensus clustering revealed that a few clusters seem to define the expression data, with 3 clusters being the minimal reasonably stable set (Fig. 2A). Consensus clustering analysis with k = 3 (Fig. 2B) shows that though the lobular cases are not evenly distributed, no cluster clearly delineates the lobular-ductal distinction. This suggests that the molecular physiology of the cells may not always present morphological features that allow an unambiguous pathological diagnosis of lobular or ductal subtypes. To better explore for gene signatures which can define these patient subsets, we collected the raw gene expression data from the selected cohorts and an initial working gene set was defined from a shrunken centroid analysis, in which 254 genes (Supplementary Table 1) were found to partition the samples with a comparable misclassification rate as when using the complete initial set of 21023 genes (Supplementary Fig. 1A). Clustering with this set of genes (Fig. 2C) revealed that even a smaller set of genes could better distinguish lobular and ductal histologic classes. An association of GO terms to these genes found lobular associated genes are more likely to be related to cell growth, while genes positively expressed in ductal tumors are more likely to regulate cell division (Fig. 2D). An examination of the top biological processes associated with these genes reinforces this functional division (Table 2, full list of significant associations in Supplementary Table 2); the processes associated with the genes with a positive correlation with ductal diagnosis were related to cell cycle control, while processes associated the genes whose expression is positively correlated with a lobular diagnosis comprised a list of diverse functions related to cell growth and metabolism.

To create a final model with a limited gene set, elastic-net regularized generalized linear models were used were (Supplementary Figure 1B), with a minimal error found when 46 genes were employed (listed in Supplementary Table 3). The chosen model building method used an extension of lasso modified regression, termed an elastic net, that penalizes the least square estimates and thus allows both continuous shrinkage and automatic variable selection simultaneously^44^. Genes and their coefficients from the model were used to define a pattern to which samples in the discovery cohorts were compared via a Pearson correlation. An optimal cutoff with a minimum misclassification error rate was determined by examining all discovery samples. Cutoff values that were statistically indistinct from the optimal cutoff were also determined via a Z test to define a ‘strict’ model, which excludes a diagnosis for samples which cannot be definitively diagnosed (Supplementary Figure 1C). As the molecular physiology between lobular and ductal subtypes may be more similar when matched for grade^45^, we also performed model building using only low grade samples (grades 1 or 2, which included 57 lobular cases out of 315 total). A similar model was derived, which shared 65% of the genes of the model derived using all samples, with a Pearson *r* of 0.90 between the models’ coefficients.

**Figure 3:**
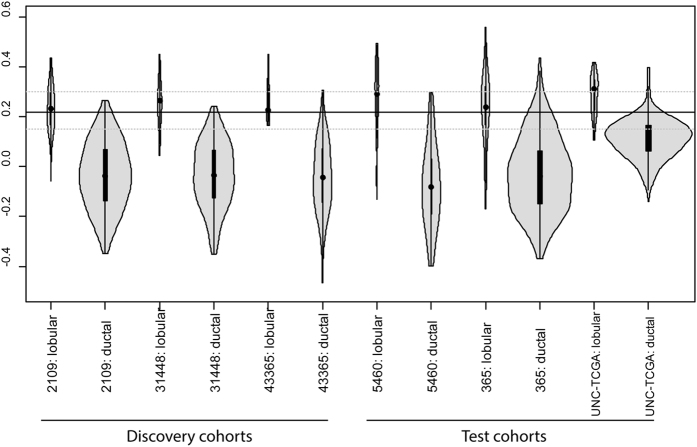
Distribution of model scores in the discovery and test cohorts. The cutoffs of the model that assigns a diagnosis to all cases is represented by a solid line, the cutoffs for the model which allows an indeterminate class are represented by dashed lines.

The distribution of model scores in the discovery and test cohorts with pathological diagnosis showed a strong association (p value < 0.0001 via T-test), with similar distributions in all cohorts (Fig. 3). The model had a 94% concordance with a pathological diagnosis in the discovery cohorts given a ductal or lobular classification, and 98% concordance when indeterminate cases were excluded (the ‘strict’ model). In the validation cohorts the concordance with pathological diagnosis was 92% when all cases were compared, and 97% when indeterminate cases were excluded (Table 3). Expression data from the UNC cohort was available both measured via RNA sequencing and microarray hybridization. Concordance between both methods was very high (94% and 98% using all cases and excluding indeterminates, respectively). As the microarray hybridization provided data on more samples, this measurement was used for subsequent analysis of these cases. The fraction of cases classified as indeterminate was 14% in the discovery cohorts, and 19% in the validation cohorts (Table 3). However the two-color hybridization arrays employed for the UNC cohort had a relatively high indeterminacy rate (32%), while the RNA sequencing data for these same cases provided only 11% indeterminates, likely reflecting a greater dynamic range in the expression data. If the RNA sequencing data is used instead of the two-color array results for the UNC cohort, the indeterminancy rate in the validation cohorts is 13%, very similar to what was observed in the discovery cohort. In this set of cohorts, the indeterminates were enriched for the histopathologically defined lobular cases, as was seen in the discovery cohort. As it is known that lobular and ductal tumors are both diverse entities^14^,^39^, this difference may only reflect the greater number of ductal cases available for modeling.

The cadherin CDH1 is often used to aid in diagnosis of lobular carcinoma, as lobular breast tumors generally exhibit low expression of this gene related to inactivating mutations, which are found in over half of lobular tumors^46^, though are also present in some ductal tumors^47^. Low expression of CDH1 is therefore suggestive of a lobular diagnosis, but not indicitve^48^,^49^. We compared the model predictions to CDH1 expression in the validation cohorts. CDH1 expression was compared between cohorts by mean centering and scaling the gene expression. As seen in other studies, ductal tumors exhibited a wide range of CDH1 expression values, while lobular tumors were generally low (Supplementary Figure S2A). A mixture model^50^ was applied to determine negative expression values for CDH1, which found 45% of lobular tumors as exhibiting low expression. Using CDH1 median value as a cutoff allowed for the correct diagnosis of 88% of lobular cases, but 52% of ductal cases were misdiagnosed (CDH1 first quartile as a cutoff allowed for 76% agreement with lobular diagnosis, and 77% agreement with ductal). The model presented in this study, which includes CDH1, allowed a range of CDH1 expression in both diagnoses (Supplementary Fig. S2B), as CDH1 expression alone is an inadequate diagnostic measure.

**Figure 4:**
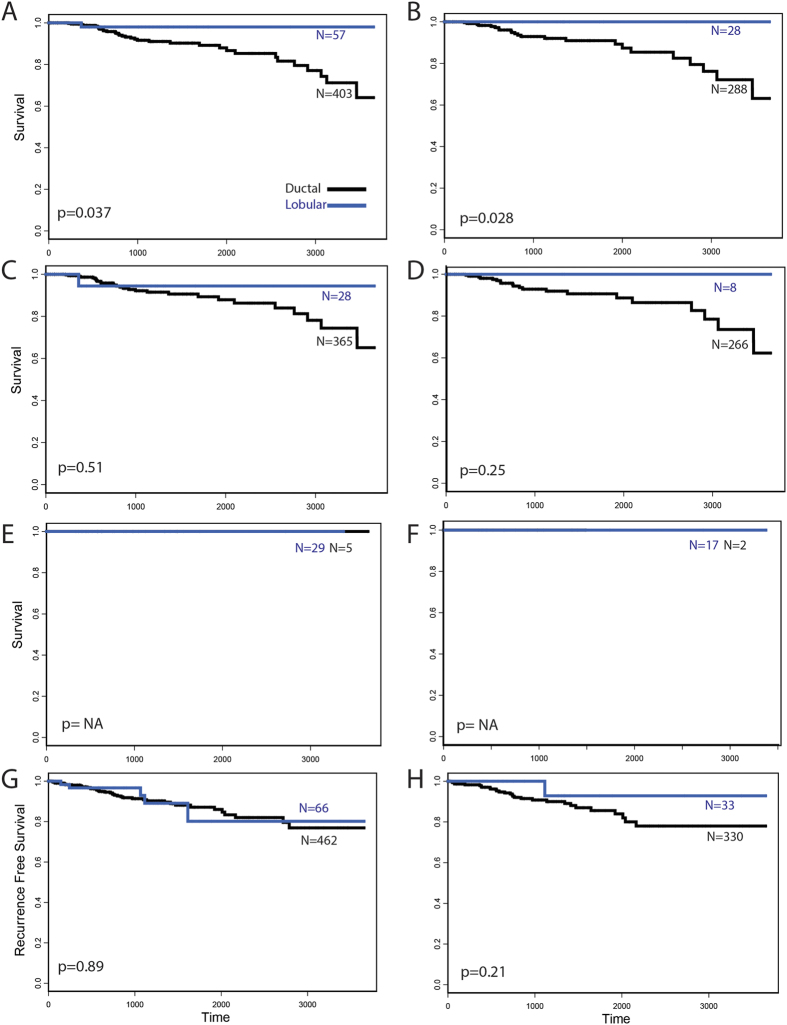
Kaplan Meier plots. (**A**) 10 year survival, all cases, all predictions and (**B**) strict predictions; (**C**) 10 year survival, pathologically-defined ductal cases, all predictions and (**D**) strict predictions; (**E**) 10 year survival, pathologically-defined lobular cases, all predictions and (**F**) strict predictions; (**G**) 10 year recurrence, all cases, all predictions and (**H**) strict predictions.

Grade and tumor type are not completely independent features, ductal tumors tend to have a preponderance of higher grade cases while lobular tumors are often moderately differentiated or grade 2. This is observed in the samples studied here (Table 4). The pathological diagnosis has a greater association with grade than does the predicted subtype (t value of −5.29; 95%CI: −7.26, −3.34 vs. −3.39; 95%CI: −5.36, −1.44 via ordered logistic regression), and when pathological diagnosis and the predicted subtype are both used to model grade, only the pathological subtype retains independence. Similarly, when the predicted subtype is adjusted for grade in its association with pathologically defined tumor type via logistic regression, the model retains significance (p value < 0.001, t value of 13.49; 95%CI: 11.58, 15.51) when using all available cohorts, and also when only the validation cohort with available grade is assessed (p < 0.001, t value of 8.12; 95%CI: 6.2, 10.14). Previous studies have demonstrated that patients with lobular tumors may have improved outcome compared to ductal carcinomas, though recent studies examining lobular subtypes suggest that classical luminal lobular cancer may have worse outcomes^51^. Examination of 10 year breast cancer related survival and recurrence for the predicted tumor types in this study showed that lobular cases have significantly better survival than ductal cases at 10 years (Table 5, Fig. 4A,B). The pathologically defined tumor types also showed a significant association with outcome (p = 0.041). Importantly, pathologically defined ductal cases that the models predicted to be lobular also showed improved outcome compared to the ductal cases not discordantly classified (Fig. 4C,D). However, although the effect size of these discordant cases is similar to that observed in all cases, significance is not reached, possibly due to the relatively small number of cases discordantly diagnosed as lobular by the model, only 7% of cases overall are reclassified by the model, and only 3% when indeterminate predictions are allowed (Table 3). When corrected for stage and ER status, significance for the association of outcome with predicted subtype was not retained, though the hazard ratio was largely unaffected (HR = 0.2; 95%CI 0.03, 1.46 vs HR = 0.26; 95CI 0.03, 1.3). Very few lobular cases were discordantly diagnosed as ductal, and no difference in outcome is observed in this small subset (Fig. 4E,F). When 10 year recurrence free survival is examined, the model that excludes indeterminate cases again shows an apparent improved outcome of lobular cases (Fig. 4G,H), though the separation of the curves was not significant (ductal and lobular subsets and their relation to recurrence free survival are shown in Supplementary Figure 3).

Differential response to therapy has been observed between lobular and ductal carcinomas, with lobular cancers responding poorly to neoadjuvant chemotherapy. This therapy is usually comprised of several combined drugs, often anthracycline plus or minus a taxane. To explore how the cellular molecular physiologies of lobular and ductal phenotypes identified with the model are associated with response to compounds in cell lines, we used the library of compound sensitivity created by the Developmental Therapeutics Program at the NIH employing a panel of 60 tumor cell lines (the NCI60 panel). These cell lines were classified by our models and given a lobular score (Supplementary Table 4). This was compared to growth inhibition measurements (GI50) across 20874 compounds on the same panel of cell lines. Some compounds with known clinical utility were found to be significantly associated with the model score; furthermore, when drugs were grouped by class, either by mechanism of action or by chemical composition, then significantly different correlations were also observed (Table 6, full list of compounds included in these classes shown in Supplementary Table 5). Anthracyclines, alkylating agents, particularly those at the N-7 position of guanine, and topoisomerase 1 and 2 inhibitors showed significance, and cell lines more similar to lobular tumors were predicted to be more sensitive to these compounds.

## Discussion

The diagnosis of lobular versus other breast carcinoma types is increasingly relevant in determining appropriate clinical care, but the definition of these types remains in question, as is a clear understanding of their underlying genesis and molecular phenotypes. Differences in visual morphology, protein expression, grade, and DNA copy number and chromosomal rearrangements and mutations have all been noted as possible classifiers^14^,^15^,^16^,^17^,^19^,^20^. Additionally, lobular carcinoma may contain molecularly distinct subgroups, making a diagnosis by pathological means alone difficult^14^. An inability to formulate a definitive classifier for these breast types hinders efforts to study differential response to treatment regimens and to identify novel targeted therapies. This is of special importance to invasive lobular tumors, which have been shown to respond poorly to standard neoadjuvant chemotherapy^32^,^33^,^34^,^35^. Additionally the development of disease is distinct for each tumor type, with lobular and ductal tumors exhibiting different metastatic behaviors^10^,^26^. Inconsistent diagnosis could contribute to inter-institute variability in treatment efficacy and have profound impacts on the sample sizes needed for adequately powered clinical studies assessing associations between treatment and tumor type.

As means of measuring of gene expression become standardized and expression-based classifiers are becoming common clinical tools we developed an ILC/IDC diagnostic tool based solely on the expression of only 46 genes. Previous studies have noted expression differences between ILC and IDC^5^ as well as a tendency of lobular cases to fall into the luminal phenotype of the intrinsic breast classification^45^, however no clearly defined model has been previously proposed and validated on independent cohorts. Despite ILC and IDC diagnoses comprising the majority of histopathologically defined tumors, these definitions do not explain the majority of the molecular physiology of the tumors (Fig. 2A–C). This is consistent with other studies that suggest that development of lobular and ductal carcinomas is not strictly based on origins within the breast duct system, but instead these tumor types may develop along a shared molecular pathway^30^,^45^. Nonetheless, expression differences exist between the tumor types, and moreover, these differences reflect biological processes that are consistent with the observed clinical behavior of the tumors (Fig. 2D).

An examination of significant enrichment of biological processes associated with the reduced list of 254 genes used for the model building showed that genes whose expression was positively associated with ductal tumors were more likely to be involved in regulation of cell cycle and proliferation, while lobular tumors were more associated with response to stimuli. This is consistent with the observation that ductal tumors tend to be more aggressive and are predictive of poorer outcome^19^, although significance was lost when adjusted for stage, ER status, or both, which may also be consistent with other studies that have shown that lobular tumors can have similar or even worse prognosis than ductal tumors when lobular subtypes are assessed^51^. The 46 selected genes for the final model included E-cadherin (CDH1), which has long been recognized to be differentially expressed between lobular and ductal tumors. CDH1 expression was strongly related to histologic diagnosis, but the model allowed for greater accuracy than CDH1 alone (Supplementary Fig. 2 and Table 3). The 254 working gene list showed some overlap with other genes previously noted to be differentially expressed between ILC and IDC, such as thrombospondin 4 and insulin-like growth factor 1^5^, but the final list was largely unique. This likely reflects the complexity of the assessed phenotype.

Comparison of the model classification to pathologist diagnosis was very good. However an inherent problem with this evaluation is weighing the meaning of discrepancies with the pathological diagnosis, as a morphological diagnosis may not be able to discern underlying molecular differences when the tumor types may share very similar lineages, even if markers like CDH1 are also employed in diagnosis. A limitation of this study is that reexamination of samples with discordant molecular and pathological diagnoses cannot be performed. However the underlying molecular physiology of a tumor may not present sufficient morphological clues to allow a pathological diagnosis that represents an accurate reflection of the tumor biology^52^. Therefore comparison of clinical course and relationship to other tumor classifications are important additional criteria. In this regard, it has been noted that lobular tumors are enriched for luminal A and B subtypes (43% and 15%, respectively). Among the pathologically defined lobular tumors, 58% were luminal A or luminal B, while in the model defined lobular tumors, 72% were luminal A or B (53% and 19%, respectively), though the PAM50 intrinsic breast cancer subtype classification model contains only one gene in common with the lobular ductal model proposed here (pituitary tumor-transforming 1, PTTG1). Similarly, lobular tumors are more likely to be grade I/II than ductal tumors^45^. This was observed in the pathologically defined tumors (Table 4). Both the pathological type and model defined type were strongly associated with grade, though pathologically defined type was the most closely associated. When the association of pathologically defined tumor type and predicted subtype is adjusted for grade, the model retains significance in all available cohorts, and also when only the validation cohort with available grade is assessed. This suggests that the model is not simply modeling grade, and the histopathological diagnosis may be more swayed by the tumor’s grade than is the molecular model.

Differences in survival outcome between subtypes were also observed. Though ILC tends to grow more slowly^19^, some studies have not observed a difference in outcome^53^. However, ILC is more frequently diagnosed late, and stage-matched analysis has seen ILC being associated with a better outcome that IDC^18^, though other studies find differing results. No difference in outcome was seen between ILC and IDC when using the pathological diagnosis (data not shown), however the model-defined lobular cases had a significantly higher 10-year survival rate compared to the predicted IDC cases, though when corrected for stage and ER status, significance for the association of outcome with predicted type was not retained. The pathologically defined ductal cases that were predicted to be lobular by the model also had a very high survival rate, though the patient number was not high enough to show significance, though the hazard ratios were very similar. This suggests some cases deemed as ductal via a traditional diagnosis may contain lobular features that a pathological diagnosis may be unable to discern. Too few pathologically defined lobular cases were reassigned to determine if this is also the case for that class of tumor.

Several studies have found that ILC is less likely to respond to neoadjuvant chemotherapy in terms of pathologic complete response than IDC and therefore neoadjuvant chemotherapy is increasingly not recommended for patients with classical ILC^32^,^33^,^34^,^35^. How to treat patients with atypical or pleomorphic lobular carcinoma is not clear. A molecular model discriminating between lobular and ductal phenotypes may help guide cell line studies exploring the sensitivity of these tumor types to different compounds. The lobular-ductal model was applied to the NCI60 panel of human tumor cell lines encompassing nine different tissues of origin, and compared to growth inhibitions measurements with relevant chemical compounds in which the concentration required to inhibit cell line growth by 50% was assayed^54^. The pattern of sensitivity of some compounds showed a significant correlation with the scores for the lobular-ductal model; however of possible greater meaning, when drugs were classed by mechanism or chemical class then cell lines predicted to be more lobular like were found to be more sensitive to anthracyclines (Table 6). Similarly, topoisomerase 2 inhibitors, which is the method through which anthracyclines effect tumor growth also showed a positive correlation with a predicted lobular nature. As neoadjuvant chemotherapy in breast cancer typically consists of an anthracycline and taxane-based regimens, this is in contrast to the observed clinical behavior of lobular tumors in response to anthracyclines. This could suggest that resistance of lobular tumors to these drugs may not be due to the different biochemistry of the tumor cell types, but their differential architecture, either of the tumor itself or surrounding breast tissues, or other tumor features, such as grade. Lobular tumors tend to be lower grade, and anthracycline based neoadjuvant therapy has been found to be more effective with high grade tumors^55^.

Interestingly, agents which act through alkylating at N-7 position of guanine, which includes mustard and platinum compounds, also were predicted in this study to significantly inhibit growth of lobular like cells. Several individual platinum agents showed a high correlation (e.g. carboplatin) between sensitivity and a lobular-like phenotype (Supplementary Table 5). Wnt/β-catenin pathway activation has been seen to be associated with poor outcomes in high grade serous ovarian cancer, and Wnt/β-catenin pathway activation was a driver of platinum chemotherapy resistance in these cases^56^. Pathway analysis revealed that the classifiers used to derive the model were highly enriched for genes associated with regulation of cell proliferation (Table 2). This class of genes includes the WNT signaling pathway GO term, and several genes specifically associated with the WNT pathway were in this classification set (Supplementary Table 1). Platinum agents are typically not employed in the treatment of lobular breast cancer, however neoadjuvant platinum-based chemotherapy for the treatment of triple negative breast cancer has been found to be effective^57^. The biochemical relationships explored in this study are neither sufficient nor intended to suggest changes to clinical care. However, additional cell line assays examining the possible relationship between platinum agents and molecular physiologies identified by this model as associated with lobular tumor may help guide continuing research into better treatment avenues.

## Methods

### Gene expression datasets and processing

We selected a total of 1845 invasive breast cancer cases in six cohorts for which expression and pathological data was publically available. The cohorts were collected by the Centre de Cancérologie de Marseille, the International Genomics Consortium, the Dana Farber Cancer Institute at Harvard University, the Ligue Nationale Contre le Cancer, Paris, France and the University of North Carolina. Expression platforms used in for obtaining expression data included Affymetrix Human Genome U133 Plus 2.0 Array, Agilent 244 K microarrays, and Illumina HiSeq RNA sequencing. Lobular tumors comprised 11% of the cohort. Three cohorts were used for discovery, and three for validation (Table 1). The three cohorts used for model building were limited to just cases unambiguously diagnosed as a ductal or lobular phenotype. The use of three cohorts for discovery and model building, employing different pathologists to perform histological subtyping, provided a heterogeneously defined population, which should aid in the creation of diagnostic models representative of a consensus definition of lobular and ductal subtypes. All provided samples were used in this analysis providing that they gave a diagnosis of lobular or ductal carcinoma and had available expression data. Raw hybridization array expression data was downloaded and normalized via Robust Multi-array Averaging and was log transformed. The three discovery cohorts were mean adjusted to remove batch effects.

### Estimates of data distribution

To get an initial assessment of how molecular diversity within the cohorts related to lobular and ductal status, cluster robustness was assessed by consensus clustering using agglomerative k-means clustering with hierarchical clustering with Pearson correlation, with a maximum k = 7. The minimum number of stable clusters was assessed by plotting the cumulative distribution, defined as when an increase in cluster number does not lead to significant increase in the cumulative distribution function. Clustering was performed for both genes and samples using hierarchical clustering with an uncentered correlation similarity metric.

### Feature reduction and model building

A derivation of a minimal gene set for model building was performed by shrunken centroid analysis^58^, which fits a nearest shrunken centroid classifier to the gene expression data. Cross validation was used to estimate the error of the shrunken centroid classifier, and a centroid was chosen using the minimal gene set before classification error of lobular cancers began to increase, resulting in a working gene set of 254 genes. Elastic-net regularized linear modeling was used to identify a final model classifying the histologic types of ductal and lobular, with 10-fold cross validation performed using a binomial model to determine the tuning parameter lambda that gave a minimum mean cross-validated error, which was then used for the predictive model^44^. A final model was derived comprised of 46 genes (Supplementary Table 3). To exclude cases for which a diagnosis may not be definitively diagnosed, cutoff values that identified a range of predictions that are statistically indistinct from the optimal cutoff were also determined via a Z test. This was termed the ‘strict’ model.

### Gene annotation and model assessment

Pathway analysis of the 254 shrunken centroid defined genes was performed with Cytoscape using the ClueGO tools^59^,^60^. This analysis allows visualization of networks of the model genes and biological processes that have been grouped with kappa statistics by similar associated genes to reduce redundancy of similar processes. Enrichment of these grouped gene ontology biological processes and KEGG terms were assessed using a step down Bonferroni correction to the alpha value. In the assessment of predicted class and their association with outcome, the likelihood ratio test was used in univariate analyses, and the Wald test for multivariate models. All p values are presented as two sided, with a value of less than 0.05 being considered significant.

### Cell line sensitivity screen

The NIH Development Therapeutics Program’s (dtp.cancer.gov) NCI60 tumor cell line growth inhibition screen was used to compare the predicted lobular nature of the cell lines in the panel to sensitivity of relevant compounds. The expression data from Affymetrex U133 Plus 2.0 chips was normalized using GC-Robust Multi-array Averaging. Lobular-ductal model scores were derived and correlated with compound sensitivity using CellMiner (discover.nci.nih.gov/cellminer)^61^.

## Additional Information

**How to cite this article:** Fu, D. *et al*. Molecular Classification of Lobular Carcinoma of the Breast. *Sci. Rep.* **7**, 43265; doi: 10.1038/srep43265 (2017).

**Publisher's note:** Springer Nature remains neutral with regard to jurisdictional claims in published maps and institutional affiliations.

## Supplementary Material

Supplementary Information

## Figures and Tables

**Table 1:** Cohort descriptions.

Study ID	Discovery cohorts			Validation cohorts			
	GSE31448^62^	GSE2109^63^	E-GEOD-43365^64^	E-GEOD-5460^65^	E-MTAB-365^66^	TCGA samples^67^	
Institution	A	B	C	C	D	E	E
Platform	1	1	1	1	1	2	3
total N	**232**	**310**	**111**	**127**	**537**	**528**	**494**
**histological type**							
ductal	210	272	96	94	335	450	423
lobular	22	38	15	19	43	41	36
mixed	0	0	0	14	1	12	0
other	0	0	0	0	55	24	34
NA	0	0	0	0	103	1	1
**menopausal status**							
pre	0	0	32	0	0	124	117
post	0	0	67	0	0	325	299
peri	0	0	0	0	0	18	18
unknown	0	0	12	0	0	33	33
NA	232	310	0	127	537	28	27
**ethnicity**							
European	0	284	0	0	0	363	344
African	0	19	0	0	0	40	40
Asian	0	3	0	0	0	34	32
other	0	3	0	0	0	1	1
unknown	0	1	0	0	0	0	0
NA	232	0	111	127	537	90	77
**pT**							
T1	49	0	0	0	0	134	127
T2	113	145	0	0	0	312	292
T3	62	23	0	0	0	59	55
T4	0	22	0	0	0	20	17
Tx	0	56	0	0	0	3	3
NA	8	64	111	127	537	0	0
**pN**							
N0	104	108	0	0	139	256	237
N1	124	89	0	0	299	170	162
N2	0	33	0	0	0	62	58
N3	0	22	0	0	0	29	26
Nx	0	0	0	0	0	11	11
NA	4	58	111	127	99	0	0
**stage**							
I	0	31	0	27	0	90	84
II	0	118	0	31	0	295	277
III	0	58	0	69	0	112	104
IV	0	4	0	0	0	14	14
X	0	0	0	0	0	16	11
NA	232	99	111	0	537	1	4
**grade**							
1	35	24	21	0	47	0	0
2	78	102	54	0	271	0	0
3	114	123	36	0	199	0	0
NA	5	61	0	127	20	528	494
**ER**							
positive	129	137	93	74	407	374	374
negative	102	69	18	53	108	113	113
NA	1	104	0	0	22	41	7
**PR**							
positive	118	110	77	0	318	0	310
negative	113	94	34	0	196	0	62
NA	1	106	0	127	23	528	122
**Her2/Erbb2**							
positive	26	53	13	30	51	297	273
negative	190	137	96	97	340	61	57
NA	16	120	2	0	146	170	164
tumor size (cm, avg.)	NA	NA	2.047	2.46	NA	NA	NA
age (avg, min-max)	NA	NA	NA	NA	56.1 (29–91)	NA	NA

Institutions: A, Centre de Cancérologie de Marseille; B, International Genomics Consortium; C, Dana Farber Cancer Institute, Harvard University; D, Ligue Nationale Contre le Cancer, Paris, France; E, Univ. North Carolina. Expression platforms: 1, Affymetrix Human Genome U133 Plus 2.0 Array; 2, Agilent 244 K microarray; 3, Illumina HiSeq RNA Seq. NA: not available.

**Table 2:** Overrepresentation of GO biological processes in shrunken centroid defined gene lists.

Gene set	GO biological process	Fold Enrichment	P value
Lobular	response to organonitrogen compound	>5	8.60E-07
Lobular	response to endogenous stimulus	3.74	1.95E-06
Lobular	response to nitrogen compound	4.66	5.86E-06
Lobular	negative regulation of cell proliferation	>5	6.88E-06
Lobular	chemical homeostasis	4.43	3.59E-05
Lobular	response to chemical	2.24	4.24E-05
Lobular	response to oxygen-containing compound	3.6	4.35E-05
Lobular	regulation of cell proliferation	3.36	4.86E-05
Lobular	cellular response to endogenous stimulus	4.06	7.85E-05
Lobular	response to organic substance	2.61	2.06E-04
Ductal	mitotic cell cycle	>5	4.49E-46
Ductal	cell cycle	>5	3.05E-45
Ductal	mitotic cell cycle process	>5	5.27E-44
Ductal	cell cycle process	>5	8.85E-44
Ductal	nuclear division	>5	1.74E-38
Ductal	mitotic nuclear division	>5	1.65E-37
Ductal	organelle fission	>5	2.38E-37
Ductal	cell division	>5	4.66E-36
Ductal	mitotic cell cycle phase	>5	1.61E-23
Ductal	cell cycle phase	>5	1.94E-23

Fold enrichment and Bonferroni corrected p values are shown.

**Table 3:**
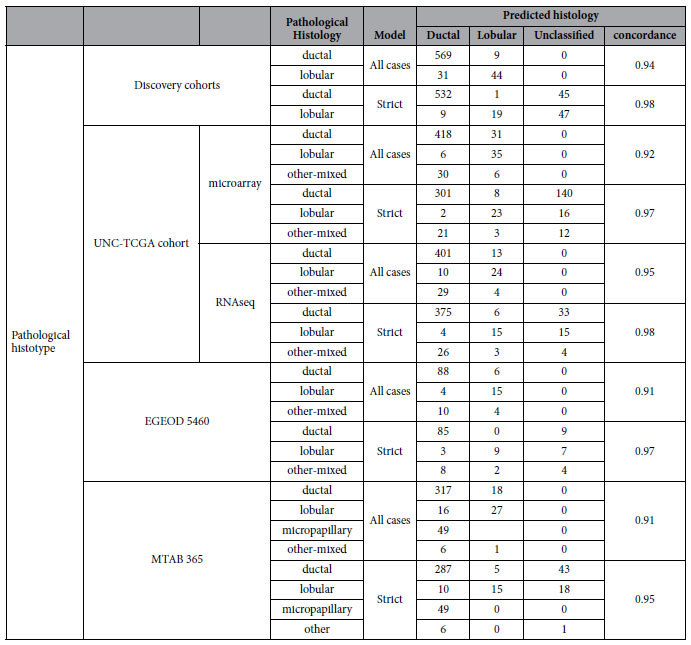
Comparison of pathological diagnoses and predicted type.

The three cohorts in the discovery set are combined, the three validation cohorts are shown separately.

**Table 4:** Comparison of diagnoses grade.

	Pathologically defined type	Molecularly defined type	Grade			
			1	2	3	Not available
GSE31448, GSE2109, E-GEOD-43365, E-MTAB-365	Ductal	ductal	93	340	395	58
		lobular	2	13	11	1
	Lobular	ductal	6	29	6	6
		lobular	11	42	12	6
	Micropapillary	ductal	2	26	16	5
		lobular	0	0	0	0
	Other or not specified	ductal	11	46	28	9
		lobular	2	9	4	1
E-MTAB-365 (validation cohort only)	Ductal	ductal	26	153	137	1
		lobular	2	9	7	0
	Lobular	ductal	0	10	4	2
		lobular	4	18	3	2
	Micropapillary	ductal	2	26	16	5
		lobular	0	0	0	0
	Other or not specified	ductal	11	46	28	9
		lobular	2	9	4	1

All cohorts with available grade are shown in the top half, and the cohort in the validation set with available grade shown below.

**Table 5:** Survival analysis.

	Pathologic Histology	Model	N	HR (95% CI)	P value
10 year BCR survival	All	all	460	0.20 (0.027, 1.46)	0.037
		strict	316	0.001 (0, >10)	0.028
	Ductal	all	393	0.55 (0.07, 4.01)	0.511
		strict	274	0.01 (0, >10)	0.245
	Lobular	all	34	1 (1, 1)	NA
		strict	19	1 (1, 1)	NA
10 year recurrence	All	all	528	0.94 (0.37, 2.34)	0.89
		strict	363	0.34 (0.05, 2.52)	0.21
	Ductal	all	450	1.16 (0.28, 4.9)	0.84
		strict	314	0.006 (0, >10)	0.29
	Lobular	all	41	>10 (0, >10)	0.5
		strict	24	1 (1, 1)	NA

Breast cancer related survival and recurrence free survival at 10 years are shown. Significant association for both models with survival is found when all histologic subtypes are combined.

**Table 6:** Cell line compound sensitivity compared to model scores.

	Mechanism of action		Model score vs GI50	# compounds	P value
**A**	Alkylating at N-7 position of guanine		0.152	46	0.000
	Topoisomerase 1 inhibitor		0.118	76	0.002
	Topoisomerase 2 inhibitor		0.128	37	0.038
**B**	**Selected drug classes**	**Mechanism of action**			
	Anthracyclines	Topoisomerase 2 inhibitor	0.169	6	0.045
	All platinum containing agents	Unknown/Alkylating agent	0.100	25	0.435
	All taxol derivatives	Tubulin affecting	0.061	22	1

The lobular-ductal model was applied to the NCI-60 panel of cell lines, and scores compared to growth inhibition measurements (GI50) across 20874 compounds. The correlation of GI50 to model scores in drug classes defined by mechanism of action (A) or chemical composition (B). A T-test of the correlations in the drug class compared to all compounds is shown, Boneferroni corrected p value.
