# Supplementary material for: Molecular Classification of Lobular Carcinoma of the Breast
Source: Sci Rep. 2017 Mar 17;7:43265. doi: 10.1038/srep43265 (PMC5355990; doi:10.1038/srep43265)

Molecular Classification of Lobular Carcinoma of the Breast

Denggang Fu[1]#, Qi Zuo[1]#, Qi Huang[1], Li Su[2], Huijun Z. Ring[1]*, Brian Z. Ring[1]*

# co-first authors; * co-communication authors

Affiliations:

[1]Institute of Genomic and Personalized Medicine, College of Life Science, Huazhong University of Science and Technology, Wuhan, Hubei, China 430074

[2]Key Laboratory of Molecular Biophysics, Ministry of Education, College of Life Science, Huazhong University of Science and Technology, Wuhan, Hubei, China 430074

Corresponding authors:

Brian Z. Ring, Institute of Genomic and Personalized Medicine, College of Life Science and Technology, Huazhong University of Science and Technology, Wuhan, Hubei, China 430074; Phone: +86 185-0305-7601; Email: bzring@hust.edu.cn

Huijun Z. Ring, Institute of Genomic and Personalized Medicine, College of Life Science and Technology, Huazhong University of Science and Technology, Wuhan, Hubei, China 430079; Phone: +86 150-1006-3688; Email: hzring@gmail.com

Supplementary Table Descriptions

Supplementary Table S1. Genes selected by shrunken centroid analysis.

Supplementary Table S2. All GO terms significantly associated with classifying gene set.

Supplementary Table S3. The genes and coefficients that comprise the model.

Supplementary Table S4. NCI60 cell lines and model scores

Supplementary Table S5. Cell line sensitivity (individual drugs)

Supplementary Figure Legends

Supplementary Figure 1. Defining limited sets of genes that classify lobular and ductal carcinoma. A) Shrunken centroid analysis. B) Further reduction with elastic-net regularized generalized linear models. C) Cutoff determination for elastic net-defined model. Error rate at all cutoffs is shown. Optimal cutoff is represented by the dashed line. Black vertical lines indicate the region in which cutoff values are statistically indistinct from the optimal.

Supplementary Figure 2. CDH1 expression compared to A) the given histologic diagnosis, and B) the model prediction.

Supplementary Figure 3 Kaplan Meier plots for 10 year recurrence. A,B) all cases, all predictions and

strict predictions; C,D) ductal cases, all predictions and strict predictions; E,F) lobular cases, all predictions and strict predictions

Gene symbol	Gene name	NCBI Entrez gene ID	model coefficient
CDH1	cadherin 1, type 1, E-cadherin (epithelial)	999	-1.32
AKAP12	A kinase (PRKA) anchor protein 12	9590	-0.86
DPP3	dipeptidyl-peptidase 3	10072	-0.81
PDCD2L	programmed cell death 2-like	84306	-0.77
TRIM59	tripartite motif containing 59	286827	-0.48
SEMA3G	sema domain, immunoglobulin domain (Ig), short basic domain, secreted, (semaphorin) 3G	56920	-0.36
FAM60A	family with sequence similarity 60, member A	58516	-0.34
SDF2L1	stromal cell-derived factor 2-like 1	23753	-0.23
PDK4	pyruvate dehydrogenase kinase, isozyme 4	5166	-0.21
ACACB	acetyl-CoA carboxylase beta	32	-0.18
ADH1B	alcohol dehydrogenase 1B (class I), beta polypeptide	125	-0.16
GPD1	glycerol-3-phosphate dehydrogenase 1 (soluble)	2819	-0.12
PPARG	peroxisome proliferator-activated receptor gamma	5468	-0.11
CDCA4	cell division cycle associated 4	55038	-0.09
IGFBP6	insulin-like growth factor binding protein 6	3489	-0.07
OGN	osteoglycin	4969	-0.06
TENC1	tensin like C1 domain containing phosphatase (tensin 2)	23371	-0.05
SCN4B	sodium channel, voltage-gated, type IV, beta subunit	6330	-0.03
GCOM1	GRINL1A complex locus 1	145781	-0.03
ZBTB16	zinc finger and BTB domain containing 16	7704	-0.02
LDB2	LIM domain binding 2	9079	0.00
CMC2	C-x(9)-C motif containing 2	56942	0.00
RNASEH2A	ribonuclease H2, subunit A	10535	0.01
HLF	hepatic leukemia factor	3131	0.01
NUF2	NUF2, NDC80 kinetochore complex component	83540	0.02
APOD	apolipoprotein D	347	0.03
CHMP4C	charged multivesicular body protein 4C	92421	0.10
C4orf32	chromosome 4 open reading frame 32	132720	0.11
SAA1	serum amyloid A1	6288	0.11
NTN4	netrin 4	59277	0.12
ALDH1A1	aldehyde dehydrogenase 1 family, member A1	216	0.13
LAMA2	laminin, alpha 2	3908	0.14
ATP1A2	ATPase, Na+/K+ transporting, alpha 2 polypeptide	477	0.14
PTTG1	pituitary tumor-transforming 1	9232	0.16
KIF11	kinesin family member 11	3832	0.19
STIP1	stress-induced phosphoprotein 1	10963	0.25
THBS4	thrombospondin 4	7060	0.26

FAM129A	family with sequence similarity 129, member A	116496	0.32
RAN	RAN, member RAS oncogene family	5901	0.33
SESN1	sestrin 1	27244	0.39
TAC1	tachykinin, precursor 1	6863	0.40
KIF13B	kinesin family member 13B	23303	0.43
LPL	lipoprotein lipase	4023	0.44
SOCS2	suppressor of cytokine signaling 2	8835	0.45
TMEM119	transmembrane protein 119	338773	0.45
LMOD1	leiomodin 1 (smooth muscle)	25802	0.69

Suppl Table S1. The genes and coefficients that comprise the model.

Gene symbol	NCBI Entrez geneid	Name	ductal centroid score	lobular centroid score
ATP1A2	477	ATPase, Na+/K+ transporting, alpha 2 polypeptide	-0.0302	0.2429
LPL	4023	lipoprotein lipase	-0.0286	0.2297
TIMP4	7079	TIMP metallopeptidase inhibitor 4	-0.0267	0.2148
ADIPOQ	9370	adiponectin, C1Q and collagen domain containing	-0.0257	0.2063
PLIN1	5346	perilipin 1	-0.0255	0.205
AOC3	8639	amine oxidase, copper containing 3	-0.0236	0.1895
NTN4	59277	netrin 4	-0.0233	0.1875
CCL14	6358	chemokine (C-C motif) ligand 14	-0.0222	0.1783
THBS4	7060	thrombospondin 4	-0.0205	0.1644
CHRDL1	91851	chordin-like 1	-0.0204	0.1639
PDGFD	80310	platelet derived growth factor D	-0.0204	0.1576
GPAM	56701	In multiple Geneids	-0.0192	0.1541
LEP	3952	leptin	-0.0186	0.1494
LEPR	3953	leptin receptor	-0.018	0.1384
PODN	127435	podocan	-0.0177	0.1367
CD36	948	CD36 molecule (thrombospondin receptor)	-0.0176	0.1417
ZCCHC24	219654	zinc finger, CCHC domain containing 24	-0.0171	0.137
PRICKLE2	166336	prickle homolog 2 (Drosophila)	-0.017	0.1365
LOC100505614	100505614	hypothetical LOC100505614	-0.0166	0.1333
FXYD1	5348	FXYD domain containing ion transport regulator 1	-0.0165	0.1326
FABP4	2167	fatty acid binding protein 4, adipocyte	-0.0163	0.1308
MFAP4	4239	microfibrillar-associated protein 4	-0.0158	0.127
TGFBR3	7049	transforming growth factor, beta receptor III	-0.0156	0.1251
KIF13B	23303	kinesin family member 13B	-0.0155	0.1246
ITIH5	80760	inter-alpha-trypsin inhibitor heavy chain family, member 5	-0.0147	0.118

SPARCL1	8404	SPARC-like 1 (hevin)	-0.0143	0.1149
ACKR1	2532	Data not found	-0.0142	0.1142
OGN	4969	osteoglycin	-0.0141	0.1132
LAMA2	3908	laminin, alpha 2	-0.014	0.1123
CFD	1675	In multiple Geneids	-0.0139	0.1114
SCUBE2	57758	signal peptide, CUB domain, EGF-like 2	-0.0137	0.1101
LOC100286909	100286909	hypothetical protein LOC100286909	-0.0135	0.1087
C7	730	complement component 7	-0.0131	0.1055
NR2F1	7025	nuclear receptor subfamily 2, group F, member 1	-0.013	0.1046
LIFR	3977	leukemia inhibitory factor receptor alpha	-0.0127	0.0979
ITGA7	3679	integrin, alpha 7	-0.0126	0.1014
JAM2	58494	junctional adhesion molecule 2	-0.0121	0.0975
PLIN4	729359	perilipin 4	-0.012	0.0966
ADH1B	125	alcohol dehydrogenase 1B (class I), beta polypeptide	-0.0114	0.0919
CAV1	857	caveolin 1, caveolae protein, 22kDa	-0.0114	0.0915
RAI2	10742	retinoic acid induced 2	-0.0111	0.0889
SORBS1	10580	In multiple Geneids	-0.0111	0.0888
NOSTRIN	115677	nitric oxide synthase trafficking	-0.011	0.0886
CDO1	1036	cysteine dioxygenase type 1	-0.0109	0.0872
ZNF423	23090	zinc finger protein 423	-0.0103	0.0829
CCDC3	83643	coiled-coil domain containing 3	-0.0103	0.0828
ATP13A5	344905	ATPase type 13A5	-0.0102	0.0819
PPAP2A	8611	phosphatidic acid phosphatase type 2A	-0.01	0.0801
SOCS2	8835	suppressor of cytokine signaling 2	-0.0099	0.0792
LRRN4CL	221091	LRRN4 C-terminal like	-0.0099	0.0792
SAA1	6288	serum amyloid A1	-0.0096	0.0768
APOD	347	apolipoprotein D	-0.0095	0.076
INMT	9171	In multiple Geneids	-0.0094	0.0755
CIDEC	63924	cell death-inducing DFFA-like effector c	-0.0093	0.0746
PDE2A	5138	phosphodiesterase 2A, cGMP-stimulated	-0.009	0.0727

IGF1	3479	insulin-like growth factor 1 (somatomedin C)	-0.0088	0.0704
FAXDC2	10826	Data not found	-0.0086	0.0689
ECM2	1842	extracellular matrix protein 2, female organ and adipocyte specific	-0.0085	0.0681
ADRA2A	150	adrenoceptor alpha 2A	-0.0084	0.0676
ABCA8	10351	ATP-binding cassette, sub-family A (ABC1), member 8	-0.0084	0.0672
RBP4	5950	retinol binding protein 4, plasma	-0.0083	0.0666
PEAR1	375033	platelet endothelial aggregation receptor 1	-0.0082	0.0661
EBF1	1879	early B-cell factor 1	-0.0082	0.0658
AQP1	358	aquaporin 1 (Colton blood group)	-0.0082	0.0658
C14orf139	na	Data not found	-0.0081	0.0651
SEMA3G	56920	sema domain, immunoglobulin domain (Ig), short basic domain, secreted, (semaphorin) 3G	-0.008	0.0642
MEOX1	4222	mesenchyme homeobox 1	-0.0079	0.0638
EMCN	51705	endomucin	-0.0079	0.0636
PDK4	5166	pyruvate dehydrogenase kinase, isozyme 4	-0.0078	0.0629
TSPAN7	7102	tetraspanin 7	-0.0077	0.0617
RBMS3	27303	RNA binding motif, single stranded interacting protein 3	-0.0076	0.0608
RBPMS	11030	RNA binding protein with multiple splicing	-0.0074	0.0593
TENC1	23371	tensin like C1 domain containing phosphatase (tensin 2)	-0.0074	0.0593
CLEC3B	7123	C-type lectin domain family 3, member B	-0.0073	0.0589
RERGL	79785	RERG/RAS-like	-0.0071	0.0568
FHL1	2273	four and a half LIM domains 1	-0.0069	0.0558
HTR2B	3357	5-hydroxytryptamine (serotonin) receptor 2B, G protein-coupled	-0.0068	0.0549
DPT	1805	dermatopontin	-0.0068	0.0548
LOC100507273	100507273	collagen alpha-4(VI) chain-like	-0.0068	0.0546
COL14A1	7373	collagen, type XIV, alpha 1	-0.0067	0.0535
LHFP	10186	lipoma HMGIC fusion partner	-0.0059	0.0477
PHYHD1	254295	phytanoyl-CoA dioxygenase domain containing 1	-0.0059	0.0472
G0S2	50486	G0/G1 switch 2	-0.0057	0.0456
SHE	126669	Src homology 2 domain containing E	-0.0056	0.0446

CFH	3075	complement factor H	-0.005	0.0401
GCOM1	145781	GRINL1A complex locus 1	-0.005	0.04
GNG11	2791	guanine nucleotide binding protein (G protein), gamma 11	-0.0049	0.0397
TAC1	6863	tachykinin, precursor 1	-0.0048	0.0385
ACACB	32	acetyl-CoA carboxylase beta	-0.0047	0.0374
SDPR	8436	serum deprivation response	-0.0046	0.0373
BTG2	7832	BTG family, member 2	-0.0046	0.0372
SOWAHA	134548	Data not found	-0.0046	0.0369
TNMD	64102	tenomodulin	-0.0044	0.0353
BTNL9	153579	butyrophilin-like 9	-0.004	0.0322
GALNTL15	117248	Data not found	-0.0038	0.0308
C4orf32	132720	chromosome 4 open reading frame 32	-0.0038	0.0306
ZBTB16	7704	zinc finger and BTB domain containing 16	-0.0038	0.0303
SLIT2	9353	slit homolog 2 (Drosophila)	-0.0036	0.0293
MUSTN1	389125	musculoskeletal, embryonic nuclear protein 1	-0.0036	0.0288
FMOD	2331	fibromodulin	-0.0035	0.0284
FAM129A	116496	family with sequence similarity 129, member A	-0.0035	0.0277
TMEM119	338773	transmembrane protein 119	-0.0035	0.0277
PRELP	5549	proline/arginine-rich end leucine-rich repeat protein	-0.0034	0.0277
COX7A1	1346	cytochrome c oxidase subunit VIIa polypeptide 1 (muscle)	-0.0034	0.0273
SCN4B	6330	sodium channel, voltage-gated, type IV, beta subunit	-0.0034	0.0272
PPARG	5468	peroxisome proliferator-activated receptor gamma	-0.0031	0.025
ACVR1C	130399	activin A receptor, type IC	-0.003	0.0242
ALDH1A1	216	aldehyde dehydrogenase 1 family, member A1	-0.0029	0.0235
LDB2	9079	LIM domain binding 2	-0.0029	0.0234
CD302	9936	CD302 molecule	-0.0029	0.0234
LOC100506621	0	Data not found	-0.0029	0.0232
LMOD1	25802	leiomodin 1 (smooth muscle)	-0.0028	0.0226
GPD1	2819	glycerol-3-phosphate dehydrogenase 1 (soluble)	-0.0026	0.0212
RAPGEF3	10411	Rap guanine nucleotide exchange factor (GEF) 3	-0.0026	0.0209

MMRN2	79812	multimerin 2	-0.0023	0.0185
MEOX2	4223	mesenchyme homeobox 2	-0.0023	0.0181
LOC400043	400043	uncharacterized LOC400043	-0.0021	0.0172
SESN1	27244	sestrin 1	-0.0021	0.0168
CRY2	1408	cryptochrome circadian clock 2	-0.0021	0.0165
FAM162B	221303	family with sequence similarity 162, member B	-0.002	0.0159
CCDC178	374864	Data not found	-0.002	0.0159
TFAP2B	7021	transcription factor AP-2 beta (activating enhancer binding protein 2 beta)	-0.0018	0.0146
AKAP12	9590	A kinase (PRKA) anchor protein 12	-0.0018	0.0142
ANKRD30A	91074	ankyrin repeat domain 30A	-0.0017	0.014
SLC19A3	80704	solute carrier family 19 (thiamine transporter), member 3	-0.0016	0.013
GYG2	8908	glycogenin 2	-0.0016	0.0129
FMO2	2327	flavin containing monooxygenase 2 (non-functional)	-0.0014	0.0115
GPR146	115330	G protein-coupled receptor 146	-8.00E-04	0.0068
HLF	3131	hepatic leukemia factor	-8.00E-04	0.0064
IGFBP6	3489	insulin-like growth factor binding protein 6	-7.00E-04	0.0056
CBX7	23492	chromobox homolog 7	-7.00E-04	0.0054
CASQ2	845	calsequestrin 2 (cardiac muscle)	-5.00E-04	0.004
RBP7	116362	retinol binding protein 7, cellular	-5.00E-04	0.0038
CENPM	79019	centromere protein M	3.00E-04	-0.0027
CENPW	387103	centromere protein W	3.00E-04	-0.0021
CHEK1	1111	checkpoint kinase 1	6.00E-04	-0.005
APRT	353	adenine phosphoribosyltransferase	6.00E-04	-0.0049
MLF1IP	359815	MLF1 interacting protein	6.00E-04	-0.0046
MCM10	55388	minichromosome maintenance complex component 10	8.00E-04	-0.0065
UCK2	7371	uridine-cytidine kinase 2	8.00E-04	-0.0063
MANF	7873	mesencephalic astrocyte-derived neurotrophic factor	8.00E-04	-0.0062
EXO1	9156	exonuclease 1	9.00E-04	-0.0076
PDCD2L	84306	programmed cell death 2-like	9.00E-04	-0.0073

CMC2	56942	Data not found	0.001	-0.0081
C20orf24	55969	chromosome 20 open reading frame 24	0.0012	-0.0097
MRPL13	28998	In multiple Geneids	0.0012	-0.0094
FBXO5	26271	F-box protein 5	0.0013	-0.0103
AP1S1	1174	adaptor-related protein complex 1, sigma 1 subunit	0.0015	-0.0119
MARCKSL1	65108	MARCKS-like 1	0.0016	-0.0128
SHCBP1	79801	SHC SH2-domain binding protein 1	0.0018	-0.0145
STIP1	10963	stress-induced phosphoprotein 1	0.002	-0.0163
HIST1H2BG	8339	histone cluster 1, H2bg	0.002	-0.0161
C6orf115	353263	chromosome 6 open reading frame 115	0.002	-0.0159
TRAF4	9618	TNF receptor-associated factor 4	0.0021	-0.017
MIF	4282	macrophage migration inhibitory factor (glycosylation-inhibiting factor)	0.0022	-0.0178
CDCA4	55038	cell division cycle associated 4	0.0022	-0.0178
LRP8	4042	In multiple Geneids	0.0024	-0.0196
CHMP4C	92421	charged multivesicular body protein 4C	0.0024	-0.019
DTL	51514	denticleless E3 ubiquitin protein ligase homolog (Drosophila)	0.0025	-0.0204
NCAPD2	9918	non-SMC condensin I complex, subunit D2	0.0029	-0.0237
ADRM1	11047	adhesion regulating molecule 1	0.003	-0.0245
HSPH1	10808	heat shock 105kDa/110kDa protein 1	0.0031	-0.0253
KIF11	3832	kinesin family member 11	0.0032	-0.0261
CDCA8	55143	In multiple Geneids	0.0032	-0.0257
DKC1	1736	dyskeratosis congenita 1, dyskerin	0.0035	-0.0281
KIAA0101	9768	KIAA0101	0.0036	-0.029
AURKB	9212	aurora kinase B	0.0036	-0.0288
EPT1	85465	ethanolaminephosphotransferase 1 (CDP-ethanolamine-specific)	0.0037	-0.0301
MYBL2	4605	v-myb avian myeloblastosis viral oncogene homolog-like 2	0.0038	-0.0306
PSMG1	8624	proteasome (prosome, macropain) assembly chaperone 1	0.0039	-0.0315
SLC35B1	10237	solute carrier family 35, member B1	0.004	-0.0318
DEPDC1	55635	DEP domain containing 1	0.0042	-0.0334

ISG15	9636	ISG15 ubiquitin-like modifier	0.0044	-0.0356
RAD51AP1	10635	RAD51 associated protein 1	0.0045	-0.0365
SPAG5	10615	sperm associated antigen 5	0.0045	-0.0359
TYMS	7298	thymidylate synthetase	0.0045	-0.0359
FAM64A	54478	family with sequence similarity 64, member A	0.0046	-0.0371
KIF20A	10112	kinesin family member 20A	0.0049	-0.0392
MCM2	4171	minichromosome maintenance complex component 2	0.0053	-0.0426
ZWINT	11130	ZW10 interacting kinetochore protein	0.0055	-0.0438
PNPT1	87178	polyribonucleotide nucleotidyltransferase 1	0.0056	-0.0452
KIF23	9493	kinesin family member 23	0.0056	-0.0447
RAN	5901	RAN, member RAS oncogene family	0.0057	-0.0457
PSMA7	5688	proteasome (prosome, macropain) subunit, alpha type, 7	0.0058	-0.0468
MKI67	4288	marker of proliferation Ki-67	0.0059	-0.0471
FEN1	2237	flap structure-specific endonuclease 1	0.006	-0.0482
TLCD1	116238	TLC domain containing 1	0.0061	-0.0489
ACOT7	11332	acyl-CoA thioesterase 7	0.0061	-0.0488
DPP3	10072	dipeptidyl-peptidase 3	0.0062	-0.0497
HIST1H2BH	8345	histone cluster 1, H2bh	0.0063	-0.051
SQLE	6713	squalene epoxidase	0.0064	-0.0514
GGH	8836	gamma-glutamyl hydrolase (conjugase, folylpolygammaglutamyl hydrolase)	0.0065	-0.0523
CCNB1	891	cyclin B1	0.0065	-0.0522
NCAPH	23397	non-SMC condensin I complex, subunit H	0.0066	-0.0529
EZH2	2146	enhancer of zeste 2 polycomb repressive complex 2 subunit	0.0068	-0.0543
RNASEH2A	10535	ribonuclease H2, subunit A	0.0069	-0.0551
CDCA3	83461	In multiple Geneids	0.0071	-0.0569
HMMR	3161	hyaluronan-mediated motility receptor (RHAMM)	0.0071	-0.0567
TRIM59	286827	tripartite motif containing 59	0.0072	-0.0579
GINS1	9837	GINS complex subunit 1 (Psf1 homolog)	0.0073	-0.0583
FAM60A	58516	family with sequence similarity 60, member A	0.0076	-0.0611

TK1	7083	thymidine kinase 1, soluble	0.0077	-0.0616
CDC45	8318	cell division cycle 45	0.0079	-0.0635
RACGAP1	9042	In multiple Geneids	0.008	-0.0639
NUF2	83540	NUF2, NDC80 kinetochore complex component	0.0081	-0.0649
SDF2L1	23753	stromal cell-derived factor 2-like 1	0.0082	-0.0658
CDCA5	113130	In multiple Geneids	0.0082	-0.0658
CENPE	1062	centromere protein E, 312kDa	0.0083	-0.0669
MMP1	4312	matrix metallopeptidase 1 (interstitial collagenase)	0.0085	-0.0683
THOC4	na	Data not found	0.0085	-0.0679
MELK	9833	maternal embryonic leucine zipper kinase	0.0086	-0.0693
CCNB2	9133	cyclin B2	0.0088	-0.0675
PTTG1	9232	pituitary tumor-transforming 1	0.0089	-0.0717
HMGB3	3149	high mobility group box 3	0.009	-0.0725
PNP	4860	purine nucleoside phosphorylase	0.009	-0.0724
UHRF1	29128	In multiple Geneids	0.0091	-0.0732
AURKA	6790	In multiple Geneids	0.0098	-0.079
DHCR7	1717	7-dehydrocholesterol reductase	0.0103	-0.0824
APOBEC3B	9582	In multiple Geneids	0.0106	-0.0853
NETO2	81831	neuropilin (NRP) and tolloid (TLL)-like 2	0.0106	-0.0848
LAPTM4B	55353	lysosomal protein transmembrane 4 beta	0.011	-0.0885
BIRC5	332	baculoviral IAP repeat containing 5	0.0112	-0.0901
CCNA2	890	cyclin A2	0.0113	-0.0908
NCAPG	64151	non-SMC condensin I complex, subunit G	0.0118	-0.0945
NME1	4830	NME/NM23 nucleoside diphosphate kinase 1	0.0119	-0.0955
KPNA2	3838	karyopherin alpha 2 (RAG cohort 1, importin alpha 1)	0.0124	-0.0995
DLGAP5	9787	discs, large (Drosophila) homolog-associated protein 5	0.0125	-0.1003
FOXM1	2305	forkhead box M1	0.0126	-0.1009
NUSAP1	51203	nucleolar and spindle associated protein 1	0.0127	-0.1021
KIF2C	11004	kinesin family member 2C	0.0133	-0.1064
CENPA	1058	centromere protein A	0.0136	-0.1091

CDKN3	1033	cyclin-dependent kinase inhibitor 3	0.0137	-0.1103
CTPS1	1503	CTP synthase 1	0.014	-0.1125
TOP2A	7153	topoisomerase (DNA) II alpha 170kDa	0.0142	-0.1137
CKS2	1164	CDC28 protein kinase regulatory subunit 2	0.0146	-0.1173
BUB1B	701	BUB1 mitotic checkpoint serine/threonine kinase B	0.0148	-0.1189
NDC80	10403	NDC80 kinetochore complex component	0.0157	-0.1258
TTK	7272	TTK protein kinase	0.0158	-0.1268
CDK1	983	cyclin-dependent kinase 1	0.0162	-0.1305
ANLN	54443	anillin, actin binding protein	0.0164	-0.132
TRIP13	9319	thyroid hormone receptor interactor 13	0.0164	-0.1313
CEP55	55165	centrosomal protein 55kDa	0.0173	-0.1389
MAD2L1	4085	MAD2 mitotic arrest deficient-like 1 (yeast)	0.0177	-0.1418
UBE2S	27338	ubiquitin-conjugating enzyme E2S	0.018	-0.1442
SLC7A5	8140	solute carrier family 7 (amino acid transporter light chain, L system), member 5	0.0184	-0.1475
FAM83D	81610	family with sequence similarity 83, member D	0.0198	-0.159
KIF4A	24137	kinesin family member 4A	0.0199	-0.1597
RRM2	6241	ribonucleotide reductase M2	0.0216	-0.1731
UBE2C	11065	ubiquitin-conjugating enzyme E2C	0.0218	-0.1749
UBE2T	29089	ubiquitin-conjugating enzyme E2T (putative)	0.0221	-0.1778
TPX2	22974	TPX2, microtubule-associated	0.0224	-0.1796
HN1	51155	hematological and neurological expressed 1	0.024	-0.193
PRC1	9055	protein regulator of cytokinesis 1	0.024	-0.1926
CDH1	999	cadherin 1, type 1, E-cadherin (epithelial)	0.06	-0.4821

Suppl Table S2. Genes selected by shrunken centroid analysis.

Lobular genes (Shrunken centroid defined)

GO biological process	# in reference	# in subset	expected	Fold Enrichment	P value
response to organonitrogen compound	743	23	4.46	> 5	8.60E-07
response to endogenous stimulus	1334	30	8.01	3.74	1.95E-06
response to nitrogen compound	821	23	4.93	4.66	5.86E-06
negative regulation of cell proliferation	614	20	3.69	> 5	6.88E-06
chemical homeostasis	826	22	4.96	4.43	3.59E-05
response to chemical	3717	50	22.32	2.24	4.24E-05
response to oxygen-containing compound	1250	27	7.51	3.6	4.35E-05
regulation of cell proliferation	1438	29	8.64	3.36	4.86E-05
cellular response to endogenous stimulus	943	23	5.66	4.06	7.85E-05
response to organic substance	2359	37	14.17	2.61	2.06E-04
response to hormone	836	21	5.02	4.18	2.28E-04
homeostatic process	1269	26	7.62	3.41	2.54E-04
regulation of multicellular organismal process	2284	36	13.72	2.62	3.00E-04
negative regulation of biological process	4268	52	25.63	2.03	5.34E-04
negative regulation of cellular process	3931	49	23.61	2.08	8.03E-04
regulation of localization	2184	34	13.12	2.59	1.09E-03
response to peptide hormone	405	14	2.43	> 5	1.37E-03
small molecule metabolic process	2210	34	13.27	2.56	1.45E-03
blood circulation	290	12	1.74	> 5	1.67E-03
circulatory system process	292	12	1.75	> 5	1.80E-03
response to organic cyclic compound	728	18	4.37	4.12	3.10E-03
response to peptide	437	14	2.62	> 5	3.39E-03
cellular response to organonitrogen compound	450	14	2.7	> 5	4.79E-03
regulation of lipid metabolic process	267	11	1.6	> 5	5.71E-03
regulation of system process	406	13	2.44	> 5	9.00E-03
single-organism process	12660	101	76.03	1.33	1.01E-02
cellular response to chemical stimulus	2188	32	13.14	2.44	1.13E-02

response to stimulus	7510	71	45.1	1.57	1.47E-02
regulation of biological process	10504	89	63.08	1.41	1.48E-02
single-multicellular organism process	6003	61	36.05	1.69	1.51E-02
cellular response to nitrogen compound	499	14	3	4.67	1.59E-02
response to purine-containing compound	139	8	0.83	> 5	1.77E-02
glucose metabolic process	139	8	0.83	> 5	1.77E-02
positive regulation of transport	831	18	4.99	3.61	2.01E-02
regulation of response to wounding	369	12	2.22	> 5	2.02E-02
regulation of cellular component movement	680	16	4.08	3.92	2.72E-02
regulation of cellular localization	1125	21	6.76	3.11	2.80E-02
multicellular organismal process	6261	62	37.6	1.65	2.89E-02
regulation of biological quality	3023	38	18.15	2.09	3.51E-02
regulation of transport	1654	26	9.93	2.62	3.86E-02
carbohydrate homeostasis	157	8	0.94	> 5	4.28E-02
glucose homeostasis	157	8	0.94	> 5	4.28E-02
response to lipid	706	16	4.24	3.77	4.35E-02

Ductal genes (Shrunken centroid defined)

GO biological process	# in reference	# in subset	expected	Fold Enrichment	P value
mitotic cell cycle	783	58	4.44	> 5	4.49E-46
cell cycle	1290	67	7.31	> 5	3.05E-45
mitotic cell cycle process	716	55	4.06	> 5	5.27E-44
cell cycle process	1013	61	5.74	> 5	8.85E-44
nuclear division	440	44	2.49	> 5	1.74E-38
mitotic nuclear division	333	40	1.89	> 5	1.65E-37
organelle fission	468	44	2.65	> 5	2.38E-37
cell division	465	43	2.64	> 5	4.66E-36
mitotic cell cycle phase	294	29	1.67	> 5	1.61E-23

cell cycle phase	296	29	1.68	> 5	1.94E-23
biological phase	300	29	1.7	> 5	2.82E-23
single-organism organelle organization	1895	55	10.74	> 5	2.24E-22
mitotic M phase	221	25	1.25	> 5	3.43E-21
M phase	223	25	1.26	> 5	4.25E-21
chromosome segregation	201	24	1.14	> 5	9.53E-21
mitotic prometaphase	99	19	0.56	> 5	1.48E-19
organelle organization	2854	61	16.18	3.77	1.04E-18
regulation of cell cycle	920	36	5.22	> 5	9.48E-17
sister chromatid segregation	81	16	0.46	> 5	3.37E-16
mitotic sister chromatid segregation	73	15	0.41	> 5	2.93E-15
regulation of cell cycle process	505	27	2.86	> 5	6.97E-15
chromosome organization	865	33	4.9	> 5	1.15E-14
mitotic cell cycle phase transition	306	22	1.73	> 5	3.95E-14
cell cycle phase transition	310	22	1.76	> 5	5.17E-14
DNA metabolic process	719	29	4.08	> 5	4.90E-13
mitotic spindle organization	46	12	0.26	> 5	7.10E-13
regulation of mitotic cell cycle	460	24	2.61	> 5	1.30E-12
cellular component organization	4847	69	27.48	2.51	2.12E-12
spindle organization	92	14	0.52	> 5	2.81E-12
microtubule cytoskeleton organization	335	21	1.9	> 5	3.55E-12
cellular component organization or biogenesis	4969	69	28.17	2.45	8.17E-12
regulation of mitotic nuclear division	127	15	0.72	> 5	9.23E-12
anaphase	160	16	0.91	> 5	1.26E-11
mitotic anaphase	160	16	0.91	> 5	1.26E-11
regulation of chromosome segregation	67	12	0.38	> 5	5.86E-11
regulation of cell division	258	18	1.46	> 5	8.75E-11
cell cycle checkpoint	258	18	1.46	> 5	8.75E-11
regulation of nuclear division	152	15	0.86	> 5	1.22E-10
microtubule cytoskeleton organization involved in	27	9	0.15	> 5	6.23E-10

mitosis					
regulation of mitotic cell cycle phase transition	249	17	1.41	> 5	7.12E-10
microtubule-based process	499	22	2.83	> 5	7.84E-10
protein complex subunit organization	1382	34	7.83	4.34	1.23E-09
regulation of cell cycle phase transition	265	17	1.5	> 5	1.91E-09
protein complex assembly	933	27	5.29	> 5	1.71E-08
protein complex biogenesis	936	27	5.31	> 5	1.84E-08
anaphase-promoting complex-dependent proteasomal ubiquitin-dependent protein catabolic process	83	11	0.47	> 5	2.00E-08
DNA conformation change	221	15	1.25	> 5	2.42E-08
macromolecular complex subunit organization	2029	39	11.5	3.39	2.77E-08
single-organism process	12660	105	71.77	1.46	7.09E-08
DNA packaging	159	13	0.9	> 5	7.57E-08
single-organism cellular process	11323	99	64.19	1.54	8.94E-08
chromosome condensation	30	8	0.17	> 5	9.00E-08
DNA replication	211	14	1.2	> 5	1.79E-07
macromolecular complex assembly	1129	28	6.4	4.37	2.30E-07
spindle assembly	54	9	0.31	> 5	2.81E-07
biological_process	16510	117	93.6	1.25	3.25E-07
cytoskeleton-dependent cytokinesis	37	8	0.21	> 5	4.66E-07
negative regulation of cell cycle process	234	14	1.33	> 5	6.85E-07
mitotic spindle assembly	23	7	0.13	> 5	7.25E-07
negative regulation of cell cycle	449	18	2.55	> 5	7.79E-07
cytokinesis	95	10	0.54	> 5	1.86E-06
cellular response to DNA damage stimulus	692	21	3.92	> 5	2.91E-06
establishment of chromosome localization	48	8	0.27	> 5	3.55E-06
chromosome localization	48	8	0.27	> 5	3.55E-06
mitotic cytokinesis	29	7	0.16	> 5	3.57E-06
spindle elongation	8	5	0.05	> 5	1.08E-05
mitotic spindle elongation	8	5	0.05	> 5	1.08E-05

positive regulation of mitotic cell cycle	115	10	0.65	> 5	1.14E-05
cytoskeleton organization	830	22	4.71	4.68	1.28E-05
positive regulation of cell cycle	299	14	1.7	> 5	1.56E-05
cellular process	14078	107	79.81	1.34	2.53E-05
DNA repair	434	16	2.46	> 5	3.02E-05
modification-dependent macromolecule catabolic process	441	16	2.5	> 5	3.77E-05
cell cycle G1/S phase transition	172	11	0.98	> 5	3.97E-05
G1/S transition of mitotic cell cycle	172	11	0.98	> 5	3.97E-05
negative regulation of cell division	71	8	0.4	> 5	7.31E-05
cell cycle G2/M phase transition	142	10	0.81	> 5	8.29E-05
G2/M transition of mitotic cell cycle	142	10	0.81	> 5	8.29E-05
proteolysis involved in cellular protein catabolic process	472	16	2.68	> 5	9.68E-05
positive regulation of cell cycle process	237	12	1.34	> 5	1.01E-04
small GTPase mediated signal transduction	620	18	3.51	> 5	1.18E-04
mitotic chromosome condensation	13	5	0.07	> 5	1.19E-04
cellular protein catabolic process	488	16	2.77	> 5	1.53E-04
regulation of microtubule cytoskeleton organization	116	9	0.66	> 5	2.04E-04
modification-dependent protein catabolic process	435	15	2.47	> 5	2.29E-04
regulation of ubiquitin-protein transferase activity	118	9	0.67	> 5	2.36E-04
mitotic metaphase plate congression	31	6	0.18	> 5	2.37E-04
regulation of ligase activity	124	9	0.7	> 5	3.58E-04
cellular macromolecule catabolic process	749	19	4.25	4.47	3.76E-04
regulation of ubiquitin-protein ligase activity involved in mitotic cell cycle	90	8	0.51	> 5	4.46E-04
positive regulation of cell division	128	9	0.73	> 5	4.68E-04
protein catabolic process	534	16	3.03	> 5	5.22E-04
cellular component biogenesis	1922	31	10.9	2.84	5.22E-04
negative regulation of mitotic cell cycle	223	11	1.26	> 5	5.43E-04
metaphase plate congression	37	6	0.21	> 5	6.67E-04

establishment of organelle localization	283	12	1.6	> 5	6.83E-04
mitotic spindle midzone assembly	7	4	0.04	> 5	7.24E-04
DNA-dependent DNA replication	96	8	0.54	> 5	7.26E-04
cellular response to stress	1630	28	9.24	3.03	7.35E-04
spindle checkpoint	39	6	0.22	> 5	9.06E-04
macromolecule catabolic process	880	20	4.99	4.01	9.42E-04
cellular component assembly	1757	29	9.96	2.91	9.58E-04
organelle localization	353	13	2	> 5	9.67E-04
regulation of organelle organization	1060	22	6.01	3.66	9.70E-04
proteasome-mediated ubiquitin-dependent protein catabolic process	295	12	1.67	> 5	1.06E-03
regulation of microtubule-based process	143	9	0.81	> 5	1.18E-03
ubiquitin-dependent protein catabolic process	427	14	2.42	> 5	1.26E-03
regulation of spindle organization	21	5	0.12	> 5	1.27E-03
proteasomal protein catabolic process	302	12	1.71	> 5	1.36E-03
single-organism metabolic process	4302	49	24.39	2.01	1.72E-03
regulation of mitotic sister chromatid separation	44	6	0.25	> 5	1.83E-03
response to stimulus	7510	70	42.58	1.64	1.83E-03
regulation of chromosome organization	199	10	1.13	> 5	1.85E-03
cell proliferation	666	17	3.78	4.5	1.88E-03
spindle midzone assembly	9	4	0.05	> 5	1.96E-03
regulation of mitotic sister chromatid segregation	45	6	0.26	> 5	2.08E-03
regulation of sister chromatid segregation	45	6	0.26	> 5	2.08E-03
regulation of mitotic metaphase/anaphase transition	45	6	0.26	> 5	2.08E-03
nucleotide biosynthetic process	202	10	1.15	> 5	2.12E-03
nucleoside phosphate biosynthetic process	203	10	1.15	> 5	2.22E-03
regulation of metaphase/anaphase transition of cell cycle	46	6	0.26	> 5	2.36E-03
positive regulation of mitotic nuclear division	46	6	0.26	> 5	2.36E-03
nucleoside biosynthetic process	115	8	0.65	> 5	2.82E-03

histone phosphorylation	25	5	0.14	> 5	2.97E-03
glycosyl compound biosynthetic process	116	8	0.66	> 5	3.00E-03
negative regulation of mitotic cell cycle phase transition	160	9	0.91	> 5	3.00E-03
regulation of transcription involved in G1/S transition of mitotic cell cycle	26	5	0.15	> 5	3.60E-03
mitotic cell cycle checkpoint	169	9	0.96	> 5	4.71E-03
organelle assembly	407	13	2.31	> 5	4.77E-03
response to stress	3492	42	19.8	2.12	4.92E-03
negative regulation of cell cycle phase transition	170	9	0.96	> 5	4.94E-03
regulation of proteolysis involved in cellular protein catabolic process	280	11	1.59	> 5	5.07E-03
protein localization to chromosome, centromeric region	12	4	0.07	> 5	6.12E-03
negative regulation of nuclear division	57	6	0.32	> 5	8.13E-03
regulation of cellular protein catabolic process	296	11	1.68	> 5	8.67E-03
positive regulation of nuclear division	58	6	0.33	> 5	8.99E-03
regulation of protein ubiquitination	238	10	1.35	> 5	9.22E-03
antigen processing and presentation of exogenous peptide antigen via MHC class II	93	7	0.53	> 5	9.25E-03
pyrimidine nucleoside biosynthetic process	32	5	0.18	> 5	9.89E-03
protein localization to chromosome	32	5	0.18	> 5	9.89E-03
antigen processing and presentation of peptide antigen via MHC class II	95	7	0.54	> 5	1.06E-02
DNA strand elongation involved in DNA replication	34	5	0.19	> 5	1.33E-02
antigen processing and presentation of peptide or polysaccharide antigen via MHC class II	99	7	0.56	> 5	1.39E-02
spindle stabilization	4	3	0.02	> 5	1.42E-02
regulation of exit from mitosis	15	4	0.09	> 5	1.47E-02
regulation of transferase activity	864	18	4.9	3.67	1.48E-02
DNA strand elongation	36	5	0.2	> 5	1.75E-02

regulation of protein modification by small protein conjugation or removal	256	10	1.45	> 5	1.76E-02
pyrimidine-containing compound biosynthetic process	40	5	0.23	> 5	2.91E-02
proteolysis	1134	20	6.43	3.11	4.63E-02
positive regulation of exit from mitosis	6	3	0.03	> 5	4.74E-02

Suppl Table S3. All GO terms significantly associated with classifying gene set.

**cell line**	**Model score**
BR:BT_549	0.04
BR:HS578T	0.15
BR:MCF7	-0.24
BR:MDA_MB_231	0.01
BR:T47D	-0.21
CNS:SF_268	-0.08
CNS:SF_295	0.00
CNS:SF_539	0.04
CNS:SNB_19	0.06
CNS:SNB_75	0.05
CNS:U251	0.01
CO:COLO205	-0.22
CO:HCC_2998	-0.18
CO:HCT_116	-0.29
CO:HCT_15	-0.21
CO:HT29	-0.22
CO:KM12	-0.14
CO:SW_620	-0.28
LC:A549	-0.23
LC:EKVX	-0.22
LC:HOP_62	-0.13
LC:HOP_92	-0.01
LC:NCI_H226	-0.11
LC:NCI_H23	-0.08
LC:NCI_H322M	-0.24
LC:NCI_H460	-0.04
LC:NCI_H522	-0.07
LE:CCRF_CEM	-0.04
LE:HL_60	-0.01
LE:K_562	0.06
LE:MOLT_4	-0.07
LE:RPMI_8226	0.01
LE:SR	0.07
ME:LOXIMVI	-0.08
ME:M14	-0.03
ME:MALME_3M	-0.20
ME:MDA_MB_435	0.02
ME:MDA_N	NA
ME:SK_MEL_2	-0.03
ME:SK_MEL_28	-0.09
ME:SK_MEL_5	-0.10
ME:UACC_257	-0.24
ME:UACC_62	0.02

OV:IGROV1	-0.01
OV:NCI_ADR_RES	-0.08
OV:OVCAR_3	-0.25
OV:OVCAR_4	-0.16
OV:OVCAR_5	-0.11
OV:OVCAR_8	-0.02
OV:SK_OV_3	-0.10
PR:DU_145	-0.21
PR:PC_3	-0.29
RE:786_0	0.02
RE:A498	0.05
RE:ACHN	-0.14
RE:CAKI_1	-0.12
RE:RXF_393	-0.07
RE:SN12C	-0.06
RE:TK_10	0.05
RE:UO_31	-0.08
All (average)	-0.09
All Breast (average)	-0.05
All CNS (average)	0.01
All Colon (average)	-0.22
All Lung cancer (average)	-0.13
All Leukemia (average)	0.00
All Melanoma (average)	-0.08
All Ovarian (average)	-0.10
All Renal (average)	-0.04
All Prostate (average)	-0.25

Suppl Table S4. NCI60 cell lines and model scores

Name	NSC number	Mechanism of Action	FDA Status	Lobular score vs GI50 across the NCI60 (correlation)
Guanazole	1895	A7	-	0.273
Thiotepa	6396	A7	FDA approved	0.15
Porfiromycin	56410	A7	-	-0.029
Fluorodopan	73754	A7	-	0.175
Streptozocin	85998	A7	FDA approved	0.17
Methyl CCNU	95441	A7	-	0.349
PCNU	95466	A7	-	0.49
Yoshi 864	102627	A7	-	0.106
Mitozolomide	104800	A7	-	0.131
Dianhydrogalactitol	132313	A7	-	0.065
Carboqone; Carbazilquinone	134679	A7	-	0.021
Piperazinedione	135758	A7	-	0.057
Asaley	167780	A7	-	0.27
Spirohydantoin Mustard	172112	A7	-	0.149
Chlorozotocin	178248	A7	-	0.379
AZQ	182986	A7	-	0.155
CHIP	256927	A7	-	-0.136
Oxaliplatin	266046	A7	FDA approved	0.074
Carboxyphthalatoplatinum	271674	A7	-	-0.147
BEN	281612	A7	Clinical trial	-0.003
Teroxirone	296934	A7	-	0.137
Hepsulfam	329680	A7	-	0.252
Clomesone	338947	A7	-	0.338
Piperazine	344007	A7	-	0.225
Cyclodisone	348948	A7	-	0.309
Mitozolomide	353451	A7	-	0.303
Tetraplatin	363812	A7	-	-0.169
Apaziquone	382456	A7	Clinical trial	-0.211
Nitrogen mustard	762	A7|AlkAg	FDA approved	-0.056
Chlorambucil	3088	A7|AlkAg	FDA approved	0.195
Melphalan	8806	A7|AlkAg	FDA approved	0.151
Triethylenemelamine	9706	A7|AlkAg	FDA approved	0.164
Pipobroman	25154	A7|AlkAg	FDA approved	0.138
Cyclophosphamide	26271	A7|AlkAg	FDA approved	0.077
Uracil mustard	34462	A7|AlkAg	FDA approved	0.196
Procarbazine	77213	A7|AlkAg	FDA approved	0.125
Ifosfamide	109724	A7|AlkAg	FDA approved	0.154
Cisplatin	119875	A7|AlkAg	FDA approved	0.211
Carboplatin	241240	A7|AlkAg	FDA approved	0.317

Carmustine	409962	A7|AlkAg	FDA approved	0.439
Nitrogen mustard	757087	A7|AlkAg	FDA approved	-0.08
Melphalan	757098	A7|AlkAg	FDA approved	0.167
Camptothecin	94600	T1	-	0.066
Camptothecin Derivative	95382	T1	-	0.305
Camptothecin	100880	T1	-	0.045
Deoxycamptothecin	105132	T1	-	0.233
Camptothecin Derivative	107124	T1	-	0.027
Methoxycamptothecin	111533	T1	-	0.203
Camptothecin Derivative	176323	T1	-	0.013
Camptothecin Derivative	249910	T1	-	0.044
Camptothecin Derivative	295499	T1	-	0.29
Camptothecin Derivative	295500	T1	-	0.309
Camptothecin Derivative	295501	T1	-	0.286
Camptothecin Derivative	302991	T1	-	0.107
Indenoisoquinoline derivative	314622	T1	-	0.195
Camptothecin Derivative	328410	T1	-	0.397
Morpholinodoxorubicin	354646	T1	-	-0.008
Camptothecin Derivative	364830	T1	-	0.024
10-aminocamptothecin	369395	T1	-	0.023
Camptothecin Derivative	374028	T1	-	0.071
Aminocamptothecin	603071	T1	-	0.109
Camptothecin Derivative	606172	T1	-	0.067
Camptothecin Derivative	606173	T1	-	0.319
Camptothecin Derivative	606497	T1	-	0.079
Camptothecin Derivative	606498	T1	-	0.376
Camptothecin Derivative	606499	T1	-	0.082
Camptothecin Derivative	606985	T1	-	-0.007
Camptothecin Derivative	606986	T1	-	0.045
Topotecan	609699	T1	FDA approved	0.079
Camptothecin Derivative	610456	T1	-	0.044
Camptothecin Derivative	610457	T1	-	0.065
Camptothecin Derivative	610458	T1	-	0.136
Camptothecin Derivative	610459	T1	-	0.057
Irinotecan	616348	T1	FDA approved	0.163
Camptothecin Derivative	618939	T1	-	0.113
Camptothecin Derivative	629971	T1	-	0.073
10,11-methylenedioxycamptothecin	634724	T1	-	0.058
Camptothecin Derivative	639174	T1	-	0.199
Camptothecin Derivative	643833	T1	-	-0.019
7-Ethyl-10-hydroxycamptothecin	673596	T1	FDA approved	0.092
Camptothecin Derivative	681632	T1	-	0.207
Camptothecin Derivative	681633	T1	-	0.198
Camptothecin Derivative	681634	T1	-	0.042
Camptothecin Derivative	681635	T1	-	0.118

Camptothecin Derivative	681636	T1	-	0.239
Camptothecin Derivative	681637	T1	-	0.161
Camptothecin Derivative	681638	T1	-	0.176
Camptothecin Derivative	681639	T1	-	0.124
Camptothecin Derivative	681640	T1	-	0.144
Camptothecin Derivative	681641	T1	-	0.167
Camptothecin Derivative	681642	T1	-	0.142
Camptothecin Derivative	681643	T1	-	0.205
Camptothecin Derivative	681644	T1	-	0.139
Camptothecin Derivative	681645	T1	-	0.198
Camptothecin Derivative	681646	T1	-	0.083
Camptothecin Derivative	683555	T1	-	0.129
Camptothecin Derivative	683556	T1	-	0.13
Camptothecin Derivative	683557	T1	-	0.13
Camptothecin Derivative	683558	T1	-	0.089
Indenoisoquinoline derivative	706744	T1	-	0.1
7-Tert-butyldimethylsilyl-10-hydroxycamptothecin	708298	T1	Clinical trial	-0.036
Camptothecin Derivative	709237	T1	-	0.074
Karenitecin	710270	T1	Clinical trial	-0.121
LMP-400	724998	T1	Clinical trial	0.178
LMP776	725776	T1	Clinical trial	-0.033
Irinotecan	728073	T1	FDA approved	0.019
Indenoisoquinoline derivative	734294	T1	-	0.013
Camptothecin Derivative	735037	T1	-	0.234
Indenoisoquinoline derivative	736202	T1	-	0.111
Indenoisoquinoline derivative	736493	T1	-	0.076
Indenoisoquinoline derivative	736624	T1	-	0.145
Indenoisoquinoline derivative	737518	T1	-	0.244
Indenoisoquinoline derivative	740268	T1	-	0.049
Indenoisoquinoline derivative	740521	T1	-	-0.109
Indenoisoquinoline derivative	740645	T1	-	0.021
Topotecan	759263	T1	FDA approved	0.077
Irinotecan	759878	T1	FDA approved	0.151
ellipticine	71795	T2	-	-0.246
Daunorubicin	82151	T2	FDA approved	0.114
Daunorubicin	83142	T2	FDA approved	0.105
Teniposide	122819	T2	FDA approved	0.216
Doxorubicin	123127	T2	FDA approved	0.167
Etoposide	141540	T2	FDA approved	0.357
M-AMSA	141549	T2	-	0.026
M-AMSA	154948	T2	-	0.072
M-AMSA	156303	T2	-	-0.022
Dexrazoxane	169780	T2	FDA approved	-0.162
Valrubicin	246131	T2	FDA approved	0.191

M-AMSA	249992	T2	-	0.137
Idarubicin	256439	T2	FDA approved	0.054
Epirubicin	256942	T2	FDA approved	0.268
Deoxydoxorubicin	267469	T2	-	0.017
N,N-Dibenzyldaunorubicin	268242	T2	-	-0.003
Menogaril	269148	T2	-	0.058
Mitoxantrone	279836	T2	FDA approved	0.309
Mitoxantrone	301739	T2	FDA approved	0.193
Amonafide	308847	T2	Clinical trial	-0.207
Bisantrene hydrochloride	337766	T2	-	0.064
CI-921: amsacrine derivative	343499	T2	-	0.375
Oxanthrazole	349174	T2	-	0.237
2-methylellpticinium	351710	T2	-	0.16
2-methylellpticinium	352299	T2	-	0.024
Anthrapyrazole derivative	355644	T2	-	0.245
ellipticine	627505	T2	-	0.145
ellipticine	637651	T2	-	0.233
2-n-methyl-6-thiaellipticinum iodide	638066	T2	-	0.269
XK-469	656889	T2	Clinical trial	0.152
XK-469	697887	T2	Clinical trial	0.187
Daunorubicin	756717	T2	FDA approved	0.133
Etoposide	757804	T2	FDA approved	0.266
M-AMSA	758423	T2	-	0.044
Teniposide	758667	T2	FDA approved	0.226
Doxorubicin	759155	T2	FDA approved	0.151
Batracylin	320846	T2|T1	Clinical trial	0.179
Colchicine	757	Tu	-	0
Colchicine Derivative	33410	Tu	-	0.134
Vinblastine	49842	Tu	FDA approved	0.091
Vincristine	67574	Tu	FDA approved	-0.014
Trityl cysteine	83265	Tu	-	-0.003
Vinblastine	90636	Tu	FDA approved	-0.092
Benzimate	109874	Tu	Clinical trial	-0.065
Paclitaxel	125973	Tu	FDA approved	0.108
Maytansine	153858	Tu	-	0.082
Thiocolchicine	186301	Tu	-	0.059
Colchicine Derivative	320301	Tu	-	0.129
Rhizoxin	332598	Tu	-	-0.03
Thiocolchicine	361792	Tu	-	0.16
Dolastatin 10	376128	Tu	Clinical trial	-0.157
Allocolchicine	406042	Tu	-	0.09
Benzenepropanoic acid	600222	Tu	-	0.109
Vinorelbine	608210	Tu	FDA approved	-0.03
Taxol derivative	608832	Tu	-	0.103
Halichondrin B	609395	Tu	-	0.062

Docetaxel	628503	Tu	FDA approved	0.078
7-Epi-10-deacetylbaccatin III	656178	Tu	-	0.125
-	658831	Tu	-	0.156
2-Debenzoyl-2-(3',5'-difluorobenzoyl)-15(16)-anhydro-11(15->1)-abeotaxol	661746	Tu	-	0.034
-	664402	Tu	-	0.054
-	666608	Tu	-	0.155
-	671867	Tu	-	0.171
-	671870	Tu	-	0.107
-	673187	Tu	-	0.051
-	673188	Tu	-	-0.017
Epothilone B	684363	Tu	Clinical trial	0.08
Eribulin mesilate	707389	Tu	FDA approved	0.063
Ixabepilone	747973	Tu	FDA approved	0.147
Colchicine	756702	Tu	-	0.307
Vinblastine	757384	Tu	FDA approved	0.055
Paclitaxel	758645	Tu	FDA approved	0.106
Vincristine	759174	Tu	FDA approved	0.182
Docetaxel	759850	Tu	FDA approved	0.176
Vinorelbine	760087	Tu	FDA approved	0.111

Suppl Table S5. Cell line sensitivity (individual drugs)

Supplementary Figure S1

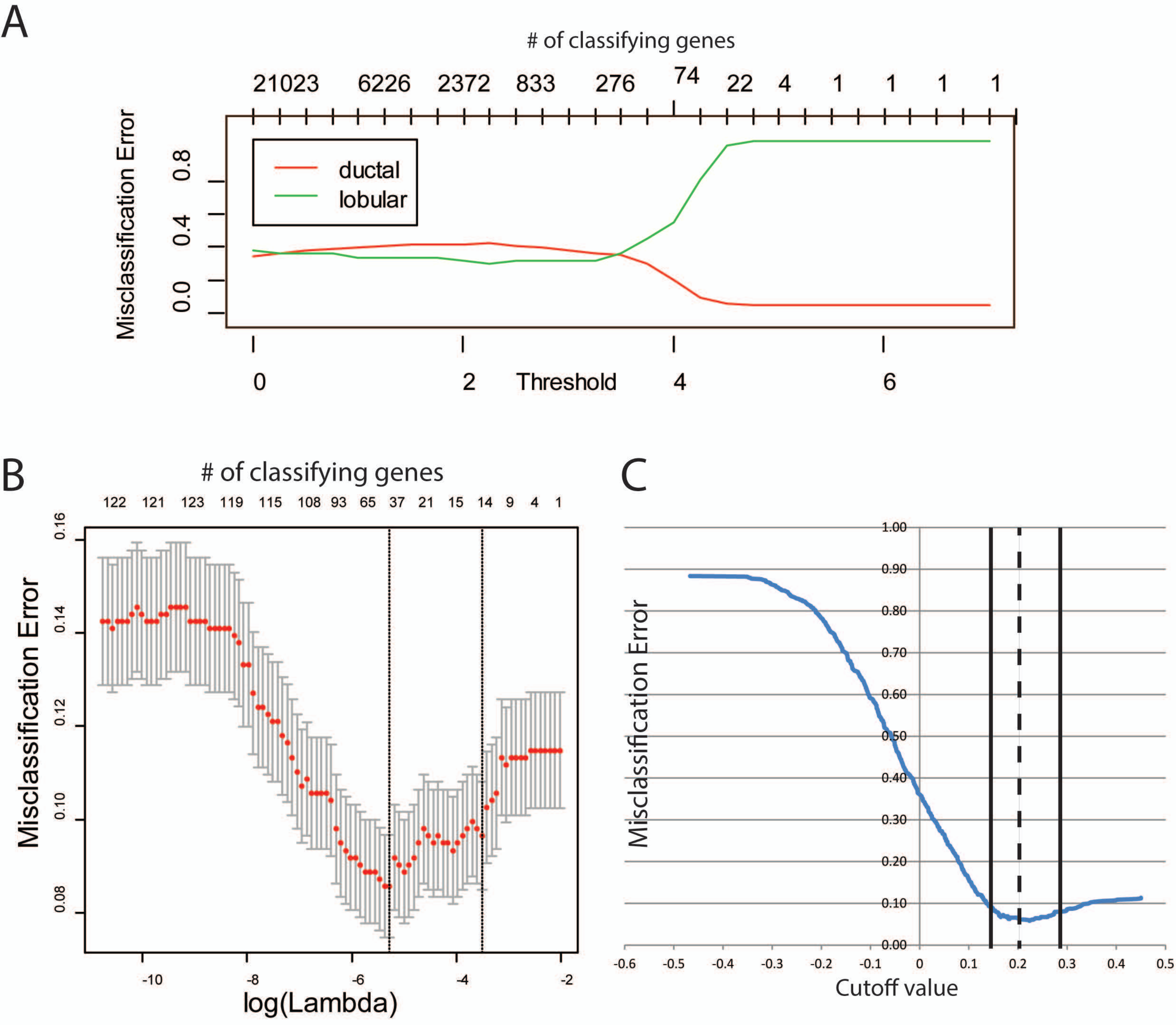

Supplementary Figure S2

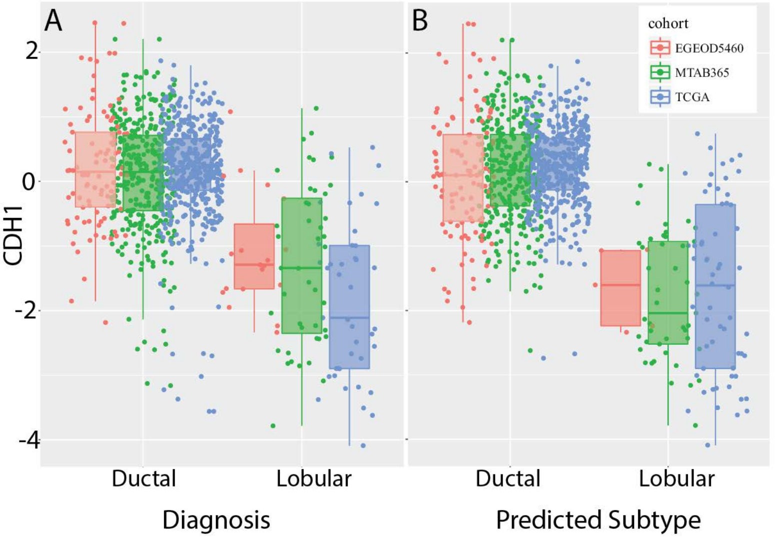

Supplementary Figure S3

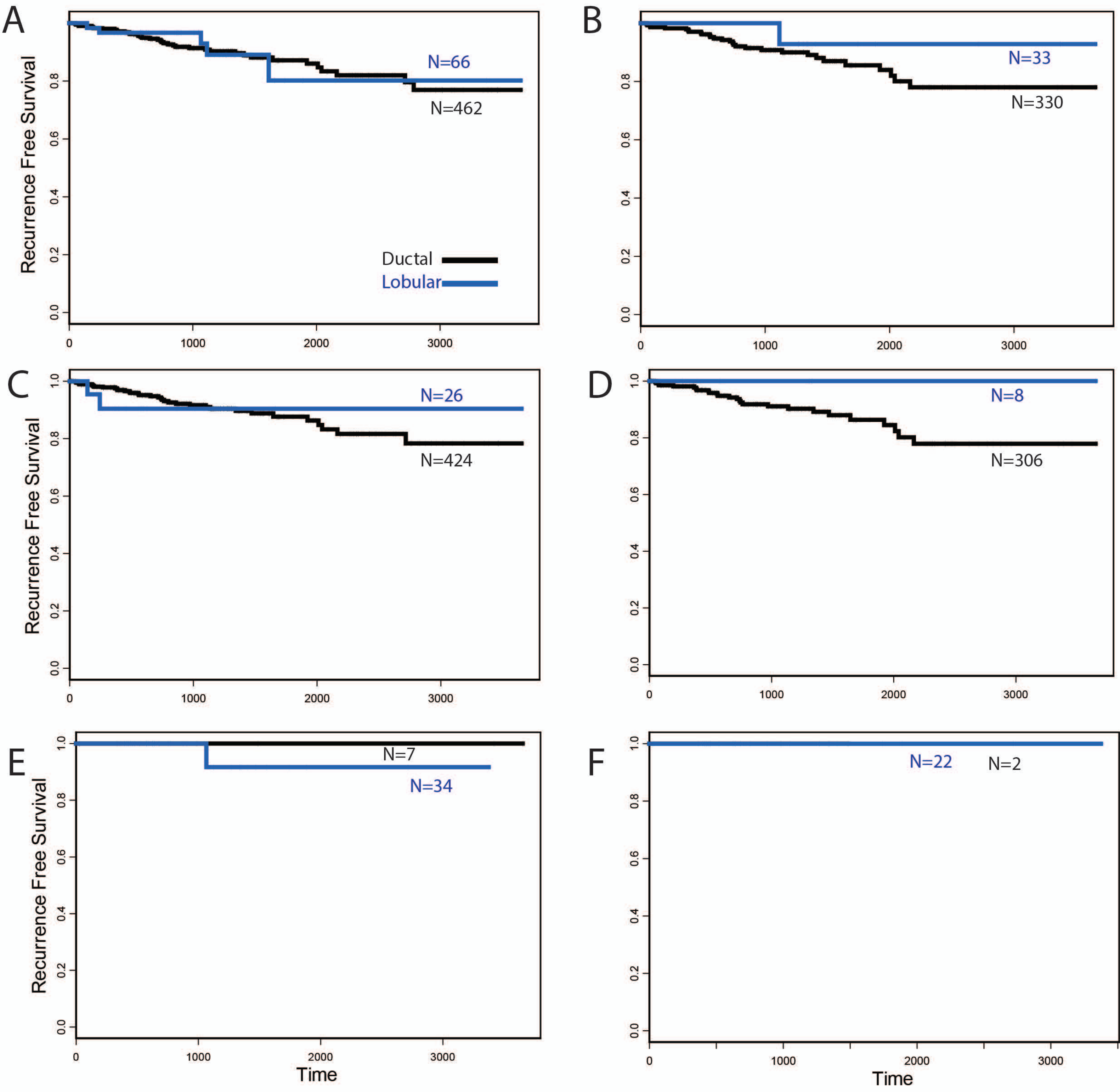

Supplement: Supplementary Information [file srep43265-s1.pdf]
